# The Role of Milk and Dairy Products in Type 2 Diabetes and Prediabetes: A Narrative Review of Randomized Trials

**DOI:** 10.3390/nu18203407

**Published:** 2026-10-17

**Authors:** Paulina Kolbe, Antonia-Therese Kietaibl, Gersina Rega-Kaun

**Affiliations:** 1 5th Medical Department with Endocrinology, Rheumatology and Acute Geriatrics, Clinic Ottakring, 1160 Vienna, Austria; paulina.kolbe@gesundheitsverbund.at (P.K.); gersina.rega-kaun@gesundheitsverbund.at (G.R.-K.); 2 Medical Faculty, Sigmund Freud University, Freudplatz 3, 1020 Vienna, Austria; 3 Verein zur Förderung der wissenschaftlichen Forschung am Wilhelminenspital Austria, Montleartstr. 37, 1160 Vienna, Austria

**Keywords:** type 2 diabetes mellitus, prediabetes, milk, dairy products, yoghurt, whey protein, glycemic control, incretin

## Abstract

**Background/Objectives**: The influence of milk and dairy products on glucose metabolism, prediabetes and type 2 diabetes is controversial. The topic has taken on new relevance following the release of the new U.S. Dietary Guidelines For Americans, recommending full-fat dairy with no added sugars on a daily basis. The purpose of this narrative review is to summarize evidence from randomized trials on the potential effects of milk and dairy products on glycemic parameters. **Methods**: For this narrative review we conducted a PubMed literature search for randomized controlled trials with combined search terms (dairy AND diabetes mellitus type 2) OR (dairy products AND diabetes mellitus type 2) OR (dairy AND pre diabetes) OR (dairy products AND pre diabetes). Eligible trials enrolled adults with prediabetes or type 2 diabetes and analyzed the influence of a milk or dairy-product intervention on glycemic markers. Included trials had full text availability and were published in English or German. **Results**: After screening for inclusion criteria, we identified 46 studies for type 2 diabetes and 11 trials for prediabetes. Findings of included studies are presented categorized by type 2 diabetes and prediabetes cohorts, by dairy intervention and by effects on acute (e.g., postprandial glucose, insulin and incretin concentrations) and chronic (e.g., hemoglobin A1c, fructosamine) glycemic control. **Conclusions**: Dairy products require a differentiated view, as their glycemic effects in people with type 2 diabetes or prediabetes vary substantially by dairy matrix, dose, timing, and specific fortification.

## 1. Introduction

Milk and dairy products have attracted interest as potential dietary modifiers of glucose metabolism [1,2]. However, they comprise a heterogeneous group of foods that differ in protein composition, carbohydrate and fat content, fermentation, and fortification [1,3]. These characteristics may influence glycemic responses and warrant consideration when evaluating dairy interventions in adults with type 2 diabetes mellitus (T2DM) or prediabetes [2,4,5].

Several dairy components provide a biological rationale for investigating these effects. Whey protein may influence postprandial glucose regulation through insulinotropic and incretin-mediated responses [4,5]. Protein form may also affect digestive kinetics: in healthy adults, micellar casein and sodium caseinate exhibited different gastric behavior and amino acid absorption patterns [6]. Calcium and vitamin D have likewise been investigated for their potential effects on glucose metabolism and insulin sensitivity [7,8,9]. These component-specific findings provide a rationale for clinical investigation, but do not establish a uniform glycemic benefit of dairy products. The key dairy components and their proposed roles in glucose metabolism are illustrated in Figure 1.

The clinical relevance of these mechanisms in adults with established T2DM or prediabetes remains insufficiently defined. In particular, it is unclear which dairy interventions improve glycemic control and whether acute postprandial responses are accompanied by sustained improvements in longer-term glycemic markers. The 2025–2030 Dietary Guidelines for Americans recommend three servings of full-fat dairy with no added sugars per day, highlighting the need for a differentiated examination of the impact of milk and dairy products on glycemic control in individuals with prediabetes or type 2 diabetes mellitus [10,11].

This narrative review, therefore, synthesizes evidence from randomized controlled trials (RCTs) evaluating milk, dairy products, and dairy-derived protein interventions in these populations. Findings are considered separately for T2DM and prediabetes and are organized by glycemic outcomes, insulin sensitivity, and endocrine responses.

## 2. Materials and Methods

This narrative review does not strictly adhere to Preferred Reporting Items for Systematic Reviews and Meta-Analyses (PRISMA guidelines); however, the PRISMA framework was applied to enhance transparency. A PRISMA-style flow diagram (Figure 2) illustrates the study identification, screening, and selection process.

### 2.1. Search Strategy and Study Selection

A literature search was conducted on PubMed on 7 April 2026 using the following search terms: (dairy AND diabetes mellitus type 2) OR (dairy products AND diabetes mellitus type 2) OR (dairy AND pre diabetes) OR (dairy products AND pre diabetes). No time restrictions were applied; articles were limited to English (100%) or German (0%) with full-text availability. Study selection and data extraction were independently performed by two authors (A.-T.K. and P.K.), with discrepancies resolved through discussion and expert input from the senior author (G.R.-K.).

Only RCTs investigating the impact of milk or dairy products on glycemic control in adults with T2DM or prediabetes were included, with no restrictions on location, duration, sample size, sex, or comparator type. Studies were excluded if participants had T1DM or gestational diabetes, lacked glycemic outcomes, or were not RCTs.

### 2.2. Narrative Synthesis

Key outcomes included fasting plasma glucose (FPG, mg/dL or mmol/L), hemoglobin A1c (HbA1c, % or mmol/mol), fructosamine, insulin sensitivity indices, postprandial glucose, plasma insulin, C-peptide, glucagon, and incretin concentrations. Statistically significant results, as reported by the original authors, were extracted alongside relevant study characteristics. Extracted data were summarized in structured tables for T2DM and prediabetes studies grouped by dairy intervention (Appendix A, Table A1 and Table A2), with key findings synthesized narratively in Section 6 and Section 7. Generative AI was used for language editing, visualization, including Figure 1, and formatting; all literature screening, data extraction, and content verification were performed by the authors.

## 3. Definition and Classification of Insulin Resistance, Prediabetes and Type 2 Diabetes Mellitus

Diabetes mellitus comprises a group of metabolic disorders characterized by hyperglycemia due to various pathogenetic reasons. Prediabetes and T2DM are classified according to the American Diabetes Association´s diagnostic criteria for FPG, HbA1c, oral glucose tolerance test (OGTT) and/or random glucose (Table 1) [12].

Prediabetes describes individuals presenting with impaired fasting glucose (IFG) and/or impaired glucose tolerance (IGT), who cannot be classified as healthy or as having overt diabetes. Prediabetes is associated with increased cardiovascular risk and progression to T2DM, particularly in the presence of metabolic syndrome components such as obesity, hypertension, and hyperlipidemia [12,13].

Insulin resistance (IR) is a pathophysiological state in which physiological insulin concentrations elicit an insufficient biological response in target tissues, representing an early manifestation of the cardiometabolic disease continuum leading to T2DM and cardiovascular disease [14]. No single diagnostic criterion exists for IR; however, surrogate measures are used in clinical practice. The Homeostasis Model Assessment of Insulin Resistance (HOMA-IR) estimates IR from fasting insulin and glucose, but primarily reflects hepatic IR [14,15]. Alternatively, metabolic syndrome criteria Adult Treatment Panel (ATP) III: any three of five components (elevated waist circumference, triglycerides ≥ 150 mg/dL, HDL-cholesterol < 40/50 mg/dL, blood pressure ≥ 130/85 mmHg, and FPG ≥ 100 mg/dL) may serve as a clinical surrogate for IR. Further clinical features suggesting IR are central/visceral obesity, acanthosis nigricans, hyperuricemia, polyendocrine metabolic ovarian syndrome, metabolic dysfunction-associated steatotic liver disease [12,16]. Lifestyle modification—including medical nutrition therapy, physical activity, and weight management—forms the foundation of treatment for both prediabetes and T2DM [13,17], making nutritional interventions a central focus of metabolic research.

## 4. Overview of Milk and Dairy Products

Milk and dairy products are complex nutritional components that consist of macronutrients (carbohydrates, fats and proteins), micronutrients (minerals and vitamins) and bioactive compounds. Whole-fat cow’s milk includes approximately 5 g of carbohydrates, 3 g of fat and 3 g of protein per 100 mL [1,18]. The key components of dairy products discussed in this section and their proposed physiological pathways relevant to glucose metabolism are provided in Figure 1.

The predominant carbohydrate in cow’s milk is lactose, a disaccharide of glucose and galactose; oligosaccharides are present only in trace amounts [1,19,20,21].

Milk fat is highly complex, comprising over 400 fatty acids—including saturated (SFA), monounsaturated (MUFA), polyunsaturated (PUFA), and trans fatty acids, and conjugated linoleic acids (CLAs)—and is organized as milk fat globules surrounded by a milk fat globule membrane (MFGM) containing polar phospholipids such as sphingomyelin and phosphatidylcholine [1,22,23,24,25].

Milk proteins consist primarily of caseins (αs1-, αs2-, β-, κ-casein) and whey proteins (β-lactoglobulin, α-lactalbumin, lactoferrin, serum albumin, and glycomacropeptide) [3,26,27]. Cow’s milk also provides calcium, phosphorus, potassium, trace elements (zinc, selenium, iron, and copper), and both fat-soluble (A, D, E, and K) and water-soluble vitamins (thiamin, riboflavin, and B12) [1,28]. However, the composition varies depending on the animals’ diet, stage of production and lactation, breed and processing method [1,18,25,29].

Dairy products are broadly classified as fermented or non-fermented. Fermented products—such as yogurt and kefir—are produced through microbial conversion of lactose to lactic acid, primarily by lactic acid bacteria (e.g., *Lactobacillus*, *Lactococcus*, *Streptococcus*), with yeasts and molds contributing in some varieties [18,30,31,32,33,34]. Non-fermented products (e.g., fresh milk, cream, ricotta) retain the original lactose content [35,36,37]. Fermentation can alter the carbohydrate content, probiotic bacteria, and bioactive compound profile, which is relevant when examining effects on glucose metabolism [18,34,38].

During fermentation of dairy products, β-galactosidase activity can generate galacto-oligosaccharides from lactose, which function as prebiotics—non-digestible compounds selectively stimulating beneficial gut bacteria [34,39,40,41].

Many fermented dairy products contain live lactic acid bacteria like *Streptococcus thermophilus* and *Lactobacillus* acting as probiotics, defined as live microorganisms conferring a health benefit to the host [40,41,42,43]. Synbiotic dairy products combine both probiotics and prebiotics (e.g., inulin) and have shown positive effects on glucose metabolism in prediabetes and T2DM. The interaction between the milk matrix, prebiotics, and probiotics is an active area of research [40,41].

Based on fat content, milk is classified as whole (≥3.25%), reduced-fat (2.0%), low-fat (1.0%), or skim (<0.5%). Reducing fat content lowers energy and saturated fat, while preserving protein, calcium, potassium, and water-soluble vitamins [1,18,44,45]. Whether low-fat milk offers cardiometabolic advantages over full-fat milk remains subject to ongoing scientific debate [11,46,47,48,49,50].

## 5. Interplay Between Milk, Dairy Products, Fat Content and Glucose Metabolism

Milk proteins—particularly whey protein, and to a lesser extent, casein—may beneficially affect glycemic control by stimulating incretin secretion (glucagon-like peptide-1, GLP-1 and glucose-dependent insulinotropic polypeptide, GIP). In vitro and animal studies suggest that bioactive peptides derived from milk-protein digestion may additionally inhibit dipeptidyl peptidase-4 in the intestine, potentially prolonging GLP-1 and GIP activity, though this has not been consistently demonstrated in humans. Milk proteins delay gastric emptying and reduce early glucose absorption, thereby attenuating postprandial glycemic excursions [5,27,51,52].

The association between dairy consumption and T2DM risk is inconclusive and product-specific [1,18,53]: meta-analyses report a small inverse association for total dairy [54,55]. Yogurt shows the most consistent inverse association, leading the Food and Drug Administration (FDA) to approve a Qualified Health Claim for yogurt and reduced T2DM risk in 2024 [54,56,57]. Evidence for milk is more heterogenous, suggesting a neutral to slightly protective effect, though very high non-fermented milk intake may increase T2DM risk [53,58,59]. Data on cheese are mixed: increased consumption was associated with higher T2DM risk in U.S. and Swedish cohorts, while the Framingham Cohort showed an inverse association with progression from prediabetes to T2DM [59,60,61]. Overall, meta-analyses and Mendelian randomization studies suggest a neutral to slightly inverse association between cheese consumption and T2DM risk [58,62,63]. Evidence regarding dairy and prediabetes is similarly heterogeneous: the Framingham Heart Study reports a 39% lower risk of new-onset prediabetes with high total dairy consumption over 12 years, whereas the Lifelines study found divergent associations by dairy type, with slightly increased risk for plain and low-fat milk, and inverse associations for fermented and skimmed dairy products [61,64,65].

The role of dairy fat content remains equally inconclusive. The AusDiab study found protective associations for high-fat dairy, high-fat milk and cheese [66], while the Fenland study associated high-fat dairy with a higher prediabetes risk and low-fat milk with a protective effect [67]. The Lifelines study reported increased prediabetes risk with of low-fat milk consumption, though this may reflect confounding by dietary patterns [65]. Among individuals with prediabetes in the Framingham population, high-fat dairy and cheese showed an inverse dose–response relationship with T2DM progression [61]. Conversely, the PREDIMED study found the strongest protective association for low-fat dairy and low-fat milk [68], while the PURE study observed the strongest inverse association with full-fat dairy and T2DM incidence [69].

## 6. Glycemic Effects of Dairy Consumption in Type 2 Diabetes

Several RCTs evaluated the long-term metabolic effects of milk and dairy-derived protein interventions in patients with established T2DM, e.g., [70,71,72,73]. Acute and preload clinical designs reveal that the structural composition and timing of dairy intake play a critical role in attenuating postprandial glucose excursions in T2DM, e.g., [74,75,76]. The glycemic-damping effects of dairy foods are largely driven by their potent insulinotropic properties and their capacity to trigger incretin hormone secretion, e.g., [74,77,78]. A comprehensive and structured overview of the study characteristics and main glycemic outcomes of all included trials is presented, grouped by dairy intervention, in Appendix A Table A1.

### 6.1. Glycemic Control and Insulin Sensitivity (HbA1c, Fructosamine, GMI, FPG, HOMA-IR)

#### 6.1.1. Hemoglobin A1c (HbA1c), Fructosamine and Glucose Management Indicator (GMI)

Effects of dairy interventions on HbA1c were heterogeneous, with favorable between-group differences reported for selected protein-based, fortified, and probiotic products, but no consistent benefit from increasing dairy consumption overall.

Increasing either low-fat or high-fat dairy intake to at least three servings daily did not affect HbA1c compared with habitual intake [79]. Similarly, comparisons of cow’s milk with soy milk and milk protein isolate with soy protein isolate showed no significant between-group differences in HbA1c [80,81].

Among protein-based dietary strategies, a whey-based high-energy breakfast produced a greater HbA1c reduction than breakfasts containing other protein sources or predominantly carbohydrates [73]. These findings contrast with trials combining whey supplementation with resistance training or high-intensity mixed-mode interval training, which found no additional HbA1c benefit over the respective exercise controls [82,83,84]. Thus, benefits observed with specific dietary strategies cannot be generalized to whey supplementation across different intervention settings.

Fortification yielded similarly inconsistent findings. A calcium-rich fat-free milk intervention within an energy-restricted diet improved HbA1c relative to the control phase, as reported in two publications from the same crossover trial [71,85]. Milk powder supplemented with inulin and resistant dextrin resulted in a smaller HbA1c increase than placebo milk powder [72]. Vitamin D-fortified yogurt drinks, with or without additional calcium, showed favorable between-group differences in HbA1c in one trial and a subsequent analysis of the same trial [86,87]. Other vitamin D-fortified yogurt interventions showed no significant HbA1c advantage in adjusted between-group comparisons [88,89,90], while unfortified low-fat milk produced a greater reduction than vitamin D-fortified milk in another trial [91]. Vitamin B-enriched non-fat yogurt, likewise, did not significantly reduce HbA1c from baseline [92]. Collectively, these findings do not establish a consistent benefit of fortification of dairy products. Moreover, comparisons between fortified and unfortified dairy products assess the effect of the added formulation, rather than dairy consumption itself.

Findings for fermented and probiotic products also varied. Selected probiotic yogurts and probiotic fermented goat’s milk improved HbA1c relative to conventional fermented controls [93,94]. However, another trial with probiotic yogurt reported a favorable between-group difference despite no significant within-group reduction in the probiotic arm, alongside an increase in the control arm [95]. Conversely, trials using *L. casei* Shirota-fermented milk or *L. helveticus*-fermented yogurt drink found no significant between-group HbA1c benefit [96,97]. A synbiotic intervention reduced HbA1c from baseline without a significant advantage over probiotic or placebo comparators [98]. These contrasting findings do not support a uniform effect across fermented or probiotic formulations.

Evidence for fructosamine and GMI was more limited, and should be considered separately from HbA1c. Probiotic fermented goat’s milk reduced fructosamine from baseline, but the corresponding between-group difference was not significant [94]. A whey preload intervention showed no significant differences in fructosamine or HbA1c compared with placebo [99]. By contrast, a dairy-enriched dietary intervention reduced continuous glucose monitoring (CGM)-derived GMI and produced a significant between-diet difference compared with a dairy-free diet [70].

Overall, some dairy-based interventions showed favorable effects on chronic glycemic markers, but the evidence does not indicate a consistent HbA1c benefit from dairy consumption. Differences in formulation, comparator, accompanying dietary or exercise interventions, and study duration limit direct comparisons, and may contribute to the heterogeneous findings. Between-group benefits must also be distinguished from within-group changes and from stabilization relative to deterioration in the control groups.

#### 6.1.2. Fasting Plasma/Serum/Blood Glucose (FPG/FSG/FBG)

No significant effects on fasting glucose compared with controls were reported for increased low-fat or high-fat dairy intake [79] or dairy-versus-soy comparisons [80,81].

Mixed results were reported for fortified dairy products. Milk powder co-supplemented with inulin and resistant dextrin reduced FPG more than placebo milk powder over 12 weeks [72]. Several vitamin D–fortified yogurt drink trials reported favorable between-group effects on fasting glucose, either by actual glucose reduction [86,89,90], or by preventing the deterioration seen in non-fortified controls [88]. Similarly, calcium-rich fat-free milk within an energy-restricted diet prevented the fasting glucose increase observed in the control phase [71,85]. In contrast, vitamin B-enriched yogurt and unfortified low-fat milk interventions did not significantly change fasting glucose from baseline [91,92]. These comparisons support a differentiated assessment of enriched products, rather than attributing their effects to the dairy matrix alone.

Probiotic yogurt containing *L. acidophilus* La5 and *B. lactis* Bb12 reduced FPG versus conventional yogurt [95], whereas other probiotic yogurt and fermented goat’s milk studies showed no between-group benefit [93,94]. An *L. helveticus*-fermented yogurt drink appeared advantageous, mainly because fasting glucose rose in the artificially acidified control group [97]. A synbiotic dairy formulation lowered fasting glucose compared with both placebo and probiotic-only treatment, while the probiotic-only group did not differ from placebo [98]. Thus, fermented dairy interventions did not consistently lower fasting glucose, and stabilization relative to a worsening comparator should be distinguished from an absolute reduction.

Favorable improvements in fasting glycemia compared with control groups were reported in trials for milk protein-based interventions: A daily dairy-protein-enriched diet reduced fasting glucose compared with a non-dairy diet [70], cysteine-rich whey protein decreased fasting glucose compared with casein [100], and a whey-based breakfast showed more pronounced effects than breakfasts based on other protein sources or carbohydrates [73]. However, adding whey to resistance or high-intensity mixed-mode training did not yield additional fasting glucose benefits beyond exercise alone [83,84], and combining resistance training with whey protein and vitamin D did not show benefits of supplementation over resistance training alone [82].

To sum up, no consistent fasting glucose benefit emerges for dairy consumption as a whole. Favorable findings occurred with selected dietary strategies, whey protein interventions, fortified products, and probiotic formulations, but were not reproduced across comparable intervention categories.

#### 6.1.3. Insulin Sensitivity and Beta-Cell Function (HOMA-IR, HOMA-β, QUICKI, Matsuda Index)

In the reviewed studies, improvements in glycemic markers did not necessarily coincide with improved insulin sensitivity, and vice versa.

Neither increased low-fat or high-fat dairy consumption nor substitution of milk protein isolate with soy protein isolate or cow´s milk with soy milk significantly altered insulin sensitivity indices [79,80,81].

Results for fortified dairy products were mixed: Vitamin D-fortified yogurt and yogurt drinks consistently showed favorable between-group effects on insulin sensitivity indices. Across three trials, vitamin D-fortified yogurt or yogurt drinks significantly improved fasting insulin, HOMA-IR, and QUICKI compared with non-fortified controls, where these markers either remained unchanged or deteriorated [86,88,89,90]. Calcium-rich fat-free milk improved beta-cell function (HOMA2-%B) compared with a low-calcium control, though HOMA2-IR did not differ between phases [85]. Milk powder co-supplemented with inulin and resistant dextrin improved both HOMA-IR and HOMA-β compared with placebo milk powder, suggesting a combined benefit on insulin sensitivity and beta-cell function [72]. In contrast, a high-calcium milk intervention showed no significant effect on HOMA2-IR compared to control [71]. Vitamin B-enriched non-fat yogurt showed no significant improvement in fasting insulin or HOMA indices [92]. Because the control groups also received dairy products, these comparisons assess the effects of added components or formulations, rather than dairy consumption itself.

Studies with probiotic yogurt, fermented goat milk, and other fermented milk products likewise reported no significant between-group improvements in fasting insulin or HOMA-IR [94,95,96,97]. In the synbiotic trial, reductions in fasting insulin and HOMA-IR occurred within the intervention group, but did not reach statistical significance in between-group comparisons versus probiotic or placebo groups [98].

Whey protein supplementation did not consistently improve insulin sensitivity indices: adding whey protein to resistance training or high-intensity mixed-mode exercise provided no significant additive benefit on HOMA-IR or insulin sensitivity compared with exercise-only controls [83,84]. A combined whey protein and vitamin D supplement showed transient within-group improvements in HOMA-IR and fasting insulin at 12 weeks, but these effects were not sustained at 24 weeks and did not differ significantly from the exercise control group at study endpoint [82]. Cysteine-rich whey protein isolate likewise produced no significant between-group differences in fasting insulin, HOMA-IR, QUICKI, or the Matsuda index compared with casein controls [100].

Taken together, selected fortified dairy products improved insulin sensitivity indices, but these effects were not consistent across dairy interventions. Glycemic markers, insulin sensitivity indices, and estimates of β-cell function should be interpreted together, as they reflect different aspects of glucose metabolism.

### 6.2. Acute Metabolic Glucose Regulation

#### 6.2.1. Nutritional Pre-Meal and Preload Strategies

Whey protein preloads reduced postprandial glucose across different doses and meal settings [74,77,101]. Differences in test meals, timing, comparators, and outcome measures prevent conclusions about a dose–response relationship across these trials. Benefits were also observed with repeated or longer-term use. Consuming whey before each main meal lowered mean glucose and postprandial excursions, and increased time in range under free-living conditions, as assessed by CGM [101]. In another trial, lower postprandial glucose and slower gastric emptying persisted after four weeks, although the between-treatment difference in peak glucose was no longer significant [99].

Postprandial responses also varied with protein formulation: compared with water, intact whey significantly reduced postprandial glucose after breakfast and lunch, whereas hydrolyzed whey only reduced the early post-breakfast response [76]. Both whey protein isolates and isolated β-lactoglobulin lowered peak interstitial glucose relative to water during home monitoring, but whey protein isolate produced lower glucose concentrations at later postprandial time points than β-lactoglobulin [102].

A sequential preload strategy, combining medium-chain triglycerides before breakfast with whey protein isolate before lunch and dinner, significantly reduced postprandial glucose and glycemic variability compared with water [103].

A whey protein preload synergistically augmented the glucose-lowering effect of the Dipeptidyl Peptidase-4 (DPP-4) inhibitor vildagliptin, significantly reducing peak postprandial glucose and overall postprandial glucose excursions beyond either intervention alone [75].

Whey preloads showed relatively consistent short-term benefits with different doses and repeated use. However, the available evidence does not establish an optimal dose, formulation, or timing strategy, and longer-term efficacy remains uncertain.

#### 6.2.2. Postprandial Effects of Co-Ingestion, Meal Replacements and Alternatives

Replacing a carbohydrate-rich breakfast with a whey-based multi-ingredient nutritional drink substantially reduced postprandial glucose excursions compared with an isocaloric rice-based meal [104]. Similarly, a high-protein breakfast containing whey protein reduced postprandial glucose compared with a high-carbohydrate control meal [73]. By contrast, adding lactose-hydrolyzed milk to bread increased postprandial glucose relative to bread and water. However, supplementing the milk with mulberry-leaf and corn-silk extracts counteracted this effect, reducing peak glucose and overall postprandial excursions compared with unsupplemented lactose-hydrolyzed milk [105]. These findings underline the importance of the accompanying ingredients and comparator meal composition when interpreting the effects of dairy co-ingestion.

Comparisons with plant-based alternatives showed mixed results: cow’s milk and almond milk consumed with oatmeal showed no significant difference in postprandial glucose [106], whereas dairy ice cream elicited lower glucose responses than a tofu-based alternative that also differed in sweeteners and other ingredients [107].

Fast-absorbing whey protein isolate and slow-absorbing micellar casein produced nearly identical postprandial glucose trajectories when consumed as part of a mixed meal, suggesting that protein absorption rate alone does not determine acute glycemic outcomes [78]. Among four distinct whey protein fractions added to a high-fat meal, no significant differences in overall postprandial glucose were observed, though fast-absorbing whey hydrolysate produced transiently higher early postprandial glucose compared with caseinoglycomacropeptide [108].

The reviewed studies showed no consistent reduction in postprandial glucose with dairy co-ingestion. Findings varied with the product, accompanying meal, control condition, and observation period, limiting generalization across dairy interventions.

### 6.3. Endocrine and Incretin Responses (Insulin, C-Peptide, Glucagon, GLP-1, GIP)

#### 6.3.1. Insulinotropic Effects and C-Peptide Secretion

Whey protein increased postprandial insulin concentrations across several preload and meal studies, but the accompanying C-peptide and secretion responses differed [74,76,77,102]. One whey preload intervention increased both insulin and C-peptide [77], whereas another increased circulating insulin partly through reduced insulin clearance, without a significant change in modelled secretion rates [74].

Comparisons between protein sources showed greater insulin, C-peptide, and proinsulin responses with whey than with casein, despite no significant difference in glucose responses [78]. Insulin responses also varied across whey fractions [102,108] and were higher after pre-meal than within-meal administration [109]. However, β-lactoglobulin increased early insulin relative to whey protein isolate without significantly different C-peptide trajectories [102]. Whey protein also produced lower peak and total C-peptide responses than a standard OGTT, while the early response did not differ [110].

Among fortified products, milk powder supplemented with inulin and resistant dextrin increased postprandial insulin more than placebo milk powder over 12 weeks [72].

Results from dairy-containing meals depended on their composition and comparator. Adding casein or a whey–soy blend to isomaltulose increased insulin response and lowered the calculated acute insulin sensitivity index [111]. Cow’s milk consumed with oatmeal significantly increased insulin iAUC compared with carbohydrate-matched almond milk [106], whereas a whey-based multi-ingredient breakfast replacement reduced glucose excursions without a significant difference in total insulin response compared with a rice-based breakfast [104].

Dairy proteins, particularly whey, frequently increased postprandial insulin, but higher concentrations did not necessarily indicate greater secretion or better glycemic control. Insulin responses should therefore be interpreted alongside C-peptide, modelled secretion, clearance, and concurrent glucose outcomes.

#### 6.3.2. Glucagon Effects

Whey preloads increased glucagon despite lowering postprandial glucose [74], and pre-meal administration produced higher early glucagon concentrations than ingesting the same dose within a meal [109].

β-Lactoglobulin elicited greater glucagon responses than whey protein isolate, while overall glucose exposure did not differ, despite lower late postprandial glucose with whey [102]. Other whey fractions showed differences at selected time points, but no significant differences in total glucagon exposure [108].

With pharmacological co-treatment, vildagliptin reduced glucagon relative to placebo under matched preload conditions, but did not eliminate the whey-associated increase [75].

In summary, glucagon responses varied with protein type, timing, and pharmacological co-treatment.

#### 6.3.3. Incretin Hormone Release (GLP-1 and GIP)

Whey preloads increased GLP-1 and/or GIP responses in several trials, although findings differed between hormones, meal setting, and comparison [74,77,109]. Both GLP-1 and GIP increased compared with placebo [74], whereas consuming whey before, rather than within, a fat-rich meal increased GIP without a significant difference in total GLP-1 [109]. In turn, a higher active GLP-1 response was observed for a whey-based multi-ingredient drink compared with a rice-based breakfast [104].

Regarding different protein fractions, there were no significant differences in GLP-1 or GIP between β-lactoglobulin and whey protein isolate [102]. Other whey fractions differed mainly in early responses: hydrolysate elicited lower GIP than isolate and higher GLP-1 than a glycomacropeptide-rich preparation [108]. Free amino acids likewise produced lower GIP responses than intact whey or casein, but still stimulated GLP-1 [78].

In a pharmacological context, whey increased total and intact GLP-1 and GIP before the meal, and its combination with vildagliptin produced higher postprandial intact hormone concentrations than either intervention alone. Notably, while vildagliptin alone suppressed total incretin secretion, co-administration of whey effectively offset this reduction by stimulating incretin release [75].

Overall, differences in study design, comparators, and outcomes measured limit direct comparisons and preclude identification of a consistent incretin response pattern across both hormones.

## 7. Glycemic Effects of Dairy Consumption in Prediabetes

Studies in adults with prediabetes examined repeated dairy consumption, protein preloads, and co-ingestion with carbohydrate-containing meals. Additional evidence came from adults with metabolic syndrome and mixed populations that included participants with prediabetes. Selected interventions showed favorable glycemic effects, but findings varied with the product, comparator, and outcome assessed [112,113,114,115,116,117,118,119,120,121]. The following sections summarize longer-term glycemic markers, fasting glucose, insulin sensitivity, postprandial glucose responses, and related hormonal outcomes. Detailed study characteristics and results are presented in Appendix A Table A2.

### 7.1. Chronic Glycemic Control and Insulin Sensitivity

#### 7.1.1. Long-Term Glycemic Markers (HbA1c, Fructosamine)

In a six-week extension of a study investigating the acute effects of different doses of a milk protein hydrolysate, daily low-dose consumption reduced HbA1c from baseline, with over half of participants reaching HbA1c levels below the prediabetes threshold [114].

Controlled yogurt trials reported favorable findings: yogurt containing heat-treated *L. plantarum* OLL2712 reduced HbA1c more than conventional yogurt [112,113]. In the later trial, this between-group difference persisted four weeks after discontinuation, but was no longer significant after eight weeks [112].

Only one study assessed fructosamine, reporting significantly lower concentrations after full-fat compared with non-fat yogurt consumption three times daily [119].

Overall, these findings suggest benefits of selected dairy products and formulations for HbA1c or fructosamine, although evidence for sustained effects remains limited.

#### 7.1.2. Fasting Glucose and Insulin Sensitivity

The effects of dairy interventions on fasting glucose and insulin sensitivity indices in individuals with prediabetes were inconsistent across studies.

While a high-dairy diet with low-fat products increased fasting insulin and insulin resistance compared with a red meat diet, fasting glucose did not differ significantly between diets. A significant diet-by-sex interaction was observed: the adverse insulin sensitivity findings were concentrated in women, who showed higher HOMA-IR and a lower Matsuda index during the high-dairy phase, whereas no significant differences in insulin sensitivity were observed in men. These findings came from a mixed population of overweight adults, including participants with impaired fasting glucose and/or impaired glucose tolerance [117].

Daily consumption of yogurt containing heat-treated *L. plantarum* OLL2712 was associated with a reduction in fasting glucose from baseline in the subgroup with impaired fasting glucose [112,113]. HOMA-IR increased in the placebo group, but remained statistically unchanged in the OLL2712 group, without a significant between-group difference [113].

### 7.2. Acute Metabolic Glucose Regulation in Prediabetes

#### 7.2.1. Pre-Meal and Preload Strategies

Protein preloads lowered postprandial glucose under selected conditions, although higher doses did not consistently provide greater glycemic benefits [114,116].

In adults with metabolic syndrome, a whey preload reduced postprandial glucose compared with water, while glucose responses did not differ significantly between the tested protein doses [116]. In participants with prediabetes, a low-dose milk protein hydrolysate preload reduced postprandial glucose iAUC, total glucose AUC, and peak glucose excursion compared with placebo, whereas the higher dose did not significantly reduce glucose iAUC relative to placebo [114].

Selected protein preloads, therefore, showed acute glycemic benefits, but higher doses did not consistently produce greater effects.

#### 7.2.2. Co-Ingestion and Beverage Studies

In participants with prediabetes, co-ingestion of dairy proteins or milk with carbohydrate loads consistently reduced postprandial glucose compared with carbohydrate alone. Adding whey protein isolate or sodium caseinate to maltodextrin reduced postprandial glucose excursions and peak glucose compared with maltodextrin alone [115]. Similarly, non-fat milk, whey, and casein all reduced glucose responses when co-ingested with a glucose load [120], and both non-fat and full-fat milk reduced glucose iAUC compared with glucose alone [121]. No significant differences in glycemic response were observed between protein sources or milk fat levels within these studies [115,120,121].

In a mixed-breakfast setting, whole and fat-free milk reduced early-phase glucose iAUC compared with water, and whole milk additionally blunted peak postprandial glucose. However, none of the milk types significantly reduced the four-hour glucose iAUC compared with water, while low-fat milk produced a lower four-hour glucose iAUC than sugar-sweetened coffee [118]. These findings were derived from pooled analyses including participants with prediabetes and healthy adults, and therefore cannot be attributed specifically to prediabetes.

### 7.3. Endocrine and Incretin Responses (Insulin, Glucagon, C-Peptide, GLP-1, GIP)

In individuals with prediabetes, dairy fat content, protein type, and dosing all influenced endocrine and incretin responses, but higher hormonal responses did not consistently translate into greater glycemic benefits.

The full-fat compared with non-fat yogurt trial reported that full-fat yogurt increased fasting GLP-1 and postprandial GIP AUC compared with non-fat yogurt, while one-hour C-peptide levels during an OGTT and total C-peptide AUC were lower after the full-fat yogurt phase [119]. In another prediabetes trial, co-ingestion of whey protein isolate or sodium caseinate with maltodextrin increased postprandial insulin, GIP and glucagon compared with maltodextrin alone, whereas GLP-1 profiles did not differ between treatments. Whey also increased C-peptide AUC, compared with maltodextrin alone [115].

In adults with metabolic syndrome, whey preloads reduced postprandial glucose but produced dose-related increases in insulin and glucagon, while only the higher dose significantly raised GLP-1; GIP remained unchanged [116]. In a mixed-breakfast comparison in a pooled sample including participants with and without prediabetes, all milk types (fat-free, low-fat, and whole milk) increased insulin iAUC compared with water, and low-fat milk elicited the highest late-phase insulin response compared with water and other beverages [118].

Taken together, dairy interventions frequently augmented postprandial insulin, glucagon, C-peptide, and/or incretin responses, but these endocrine changes varied across formulations and settings, and higher insulin or incretin responses were not consistently accompanied by greater reductions in postprandial glucose.

## 8. Discussion

This narrative review provides a structured overview of RCTs that examines the effect of milk and dairy products on glycemic control and related parameters in adults with T2DM or prediabetes and highlights the fact that isolating specific, dairy strategies such as specialized probiotic yogurt strains [93,94,113], targeted micro-nutrient fortifications [71,86,87,89], or clinically structured whey protein preloads [74,76,77,101,116] may provide clinically relevant adjunctive glycemic benefits. In T2DM, the primary clinical objective is the optimization of glycemic control with regard to cardiovascular risk and comorbidities and achievement of glycemic targets, as recommended by current guidelines [122]. In prediabetes, however, the focus shifts toward preventing disease progression and restoring normoglycemia [17].

The chronic impact of dairy consumption on long-term glycemic control is heterogenous. While several clinical trials demonstrate meaningful improvements [70,71,73,85], others report null or neutral results between groups [83,92,96,97,98]. Glycemic reductions were reported in trials employing dairy protein, probiotic and fortified dairy interventions: in patients with T2DM, improvements in HbA1c were demonstrated with a high-whey protein breakfast [73], energy-restricted diet with calcium-rich FFM [71,85], unfortified low-fat milk [91], for probiotic yogurt [93] and probiotic goat´s milk [94] interventions. Further, a reduction in GMI was shown with a dairy-enriched high-protein diet [70]. Other long-term trials found neutral or mixed effects on chronic glycemic parameters in T2DM with probiotic fermented milk [96], fermented yogurt drink [97], low-fat or full-fat dairy [79], synbiotic interventions [98], vitamin B-enriched non-fat yogurt [92], vitamin-D-fortified low-fat yogurt [88], resistance training combined with WPI [83] or whey protein plus vitamin D supplementation [82].

In individuals with prediabetes, probiotic yogurt [112,113] and pre-meal consumption of a milk protein hydrolysate [114] reduced HbA1c, while full-fat yogurt lowered fructosamine [123]. Prevention of progression to T2DM was demonstrated in one of the analyzed studies [114]. In contrast, insulin sensitivity did not improve with full-fat yogurt [123] and low-fat dairy [117] in cohorts with prediabetes. One study reported a diet-by-sex interaction, with only women showing an increase in HOMA-IR on a high-dairy diet [117], highlighting the need for sex-specific dairy interventions. The before-mentioned data goes in line with evidence from systematic reviews and meta-analyses of RCTs: in a comprehensive meta-analysis of chronic RCTs increased dairy consumption was associated with a significant, though clinically modest, overall reduction in HbA1c compared to minimal dairy intake in people without T2DM, albeit accompanied by a small increase in fasting glucose and overall uncertain clinical significance [124]. Similarly, another systematic review and meta-analysis demonstrated that increased dairy intake, especially low-fat dairy, reduced overall insulin resistance as assessed by HOMA-IR in individuals who were overweight and obese [125]. Furthermore, dose–response meta-analyses and overviews of systematic reviews confirm an inverse association between the consumption of fermented dairy products (specifically yogurt) and a smaller inverse association with dairy products in general and the incidence of type 2 diabetes [54,55,57,126,127].

The acute postprandial effects of whey protein preloading on incretin secretion and glycemic damping are fairly consistent across the identified RCTs [74,77,114,116]. The rapid appearance of intact whey peptides and free amino acids (particularly branched-chain amino acids) in the duodenum directly stimulates intestinal cells to secrete incretin hormones, though the magnitude and relative contribution of GLP-1 and GIP vary across studies [52,78,108,128,129,130]. The postprandial insulin response correlates with the appearance of circulating branched-chain amino acids [76].

Within the identified literature, clinical consistency of this strategy in T2DM is demonstrated across various protein doses [74,77,101]. The timing of administration appears relevant: consuming whey as a preload before the meal may be more effective than co-ingesting it within the meal [109,111]. The temporal gap allows circulating GLP-1 and insulin levels to rise before the dietary carbohydrates enter the bloodstream, thereby attenuating glycemic excursions at their onset [77,109]. However, the superiority of preloading over co-ingestion is not finally established: co-ingesting a small dose of whey within mixed-macronutrient meals also reduces postprandial glycemia [76]. The relative benefit of preload timing therefore remains uncertain, and warrants further direct comparison. In one identified study in patients with T2DM, isolated β-lactoglobulin elicited higher insulin and glucagon concentrations than intact whey protein, indicating that single whey fractions do not necessarily reproduce the metabolic profile of intact whey [102]. Whether these differences translate into superior postprandial glycemic control with intact whey remains unproven.

In cohorts with prediabetes or metabolic syndrome, the acute glucose-damping and endocrine-stimulating effects of whey preloads are also observed, and may serve as a potential tool for preserving early-phase beta-cell function [114,115,116]. In one study, a pre-meal whey protein load improved postprandial glucose and altered postprandial insulinemia through two mechanisms: enhanced beta-cell function and a reduction in hepatic insulin clearance, the latter contributing to higher circulating insulin without a proportional rise in secretory demand [74]. This dual mechanism may be relevant for glycemic control in T2DM and prediabetes, although its long-term impact on beta-cell preservation remains to be demonstrated in dedicated trials.

Another identified study reported that slower gastric emptying and decrease in postprandial glucose persisted after 4 weeks of regular whey protein consumption, showing that whey protein preload concepts could be a practical nutritional strategy for T2DM [99]. However, longer-term data on whey preloads strategies remain limited. A 12-week trial of a whey/guar preload reduced postprandial glucose and HbA1c in T2DM, supporting the feasibility of preload strategies, although this used a guar-containing combination product with a partly divergent GLP-1 response, so its mechanisms are not identical to the acute whey-only concept [131].

Combining dietary preloads with standard glucose-lowering medications represents a further potential strategy, particularly for incretin-based therapies. Whey protein directly stimulates enteroendocrine cells to release incretin hormones, whereas oral DPP-4-inhibitors, used as pharmacotherapy in T2DM, block the dipeptidyl peptidase-4 enzyme and, therefore, the degradation of GLP-1 and GIP. The combination of a whey preload and DPP-4-inhibition has been shown to enhance glycemic control in people with T2DM in one RCT, indicating a plausible therapeutic synergy [75]. However, there is a lack of clinical data on how dietary whey preloads interact with therapeutic incretin receptor agonists. It remains unknown whether a dietary whey preload acts additively to further optimize glycemic control and support pancreatic beta-cell function, or whether it further delays gastric emptying and potentially worsens gastrointestinal side effects (e.g., nausea, bloating). Given the widespread clinical use of these modern medications for obesity and diabetes management, this nutritional–pharmacological interaction represents a clear research gap. Future studies should therefore evaluate the potential benefits among GLP-1 receptor agonists and dual incretin mimetics in combination with whey preloads in people with T2DM.

Several methodological limitations of the reviewed literature and nature of this narrative review must be acknowledged: by restricting this review to RCTs to ensure high internal validity, important findings from long-term real-world observational cohort studies, retrospective analyses or broad epidemiological meta-analyses may have been missed. Most chronic dairy interventions have short trial durations (typically 4 to 24 weeks). The long-term durability of the reported glycemic improvements and their impact on cardiovascular endpoints or diabetes remission remain unclear. Heterogeneity of analyzed interventions in the type of dairy matrix used (e.g., whole milk, yogurt, fermented drinks, isolated whey protein formulas, bioactive peptide hydrolysates), the protein doses (e.g., 1.4 g of bioactive milk protein hydrolysate to 50 g of intact whey protein), and the timing of administration (preloads vs. co-ingestion), make direct head-to-head comparisons and generalizability difficult. Further, baseline characteristics differ across trials with confounding factors such as background diabetes pharmacotherapy (ranging from diet-controlled to insulin therapy), baseline HbA1c and the diabetes duration (not consistently reported across all trials) varying. Many dietary trials are paired with lifestyle modifications, such as nutritional counseling, calorie-restricted diets or structured exercise. This makes it difficult to isolate whether the metabolic improvements are directly attributable to the dairy intervention or are secondary to weight loss, increased physical activity, trial enrollment itself, or specific fortification (e.g., vitamin D and calcium), which further limits the generalizability of the findings.

Given the rising global incidence and prevalence of prediabetes and T2DM, there is an urgent clinical need for nutritional interventions that are highly effective, cost-efficient, and easily implementable into the daily lives of affected individuals [132]. To establish dairy-based strategies, e.g., whey protein preloading and fermented dairy consumption, as potential components of nutrition recommendation, future research should focus on longer intervention duration, to determine if the observed glycemic and metabolic benefits can be sustained over years, or if physiological adaptation and/or declining adherence attenuates their long-term efficacy. While controlled trial settings demonstrate potential efficacy, more data on regimens under real-world, free-living conditions are needed, and barriers to adherence require systematic evaluation. There is a critical shortage of gender-balanced clinical evidence: some trials in our literature search focused exclusively on men [75,76,97], only one focused on postmenopausal women [88], and another highlighted sex-specific differences in metabolic outcomes [117]. Future trials should, therefore, focus on balanced cohorts and stratify results by gender to account for the potential hormonal influences on insulin resistance, fat distribution, and glycemic regulation. Finally, there is a major gap regarding how endogenous incretin stimulation by dairy interventions interacts with modern, GLP-1- or dual GIP-/GLP-1-receptor agonists. Clinical trials are needed to evaluate whether combined nutritional and pharmacological strategies offer additive benefits or induce redundant mechanisms and compounding gastrointestinal side effects.

## 9. Conclusions

Dairy products require a differentiated view, as their glycemic effects in people with T2DM or prediabetes vary substantially by dairy matrix, dose, timing, and specific fortification. Among the identified RCTs, structured whey protein preloads emerge as a promising and mechanistically coherent strategy, consistently attenuating postprandial glycemia and stimulating endogenous incretin secretion, whereas evidence for durable improvements in chronic glycemic parameters remains more heterogeneous. Selected dairy-based approaches may thus serve as low-cost, widely available adjuncts to, not replacements for, established lifestyle and pharmacological therapy. Future research should prioritize longer, real-world, gender-balanced trials before differentiated dairy-based strategies can be firmly integrated into evidence-based nutritional recommendations.

## Figures and Tables

**Figure 1:**
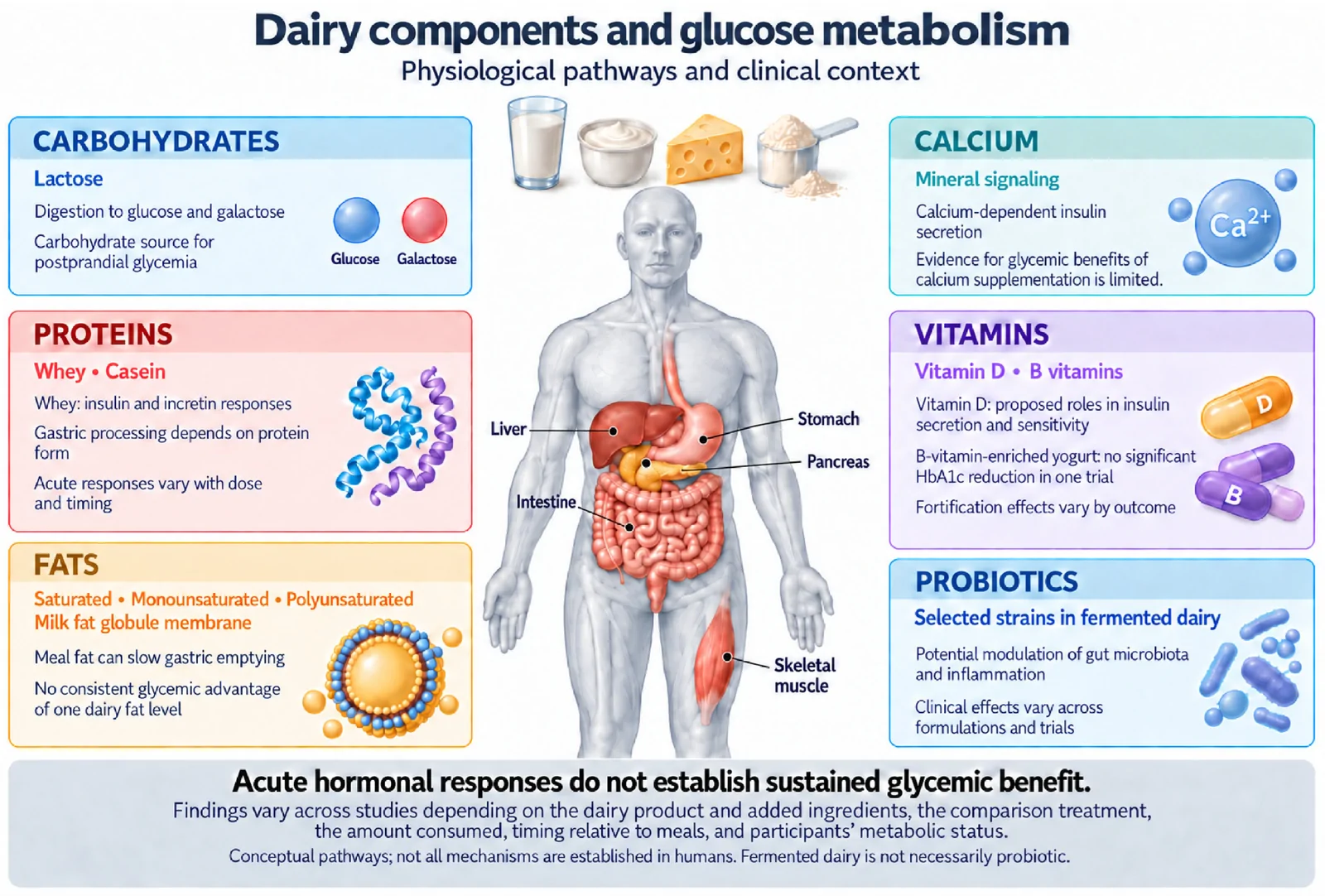
Key dairy components and their proposed roles in glucose metabolism.

**Figure 2:**
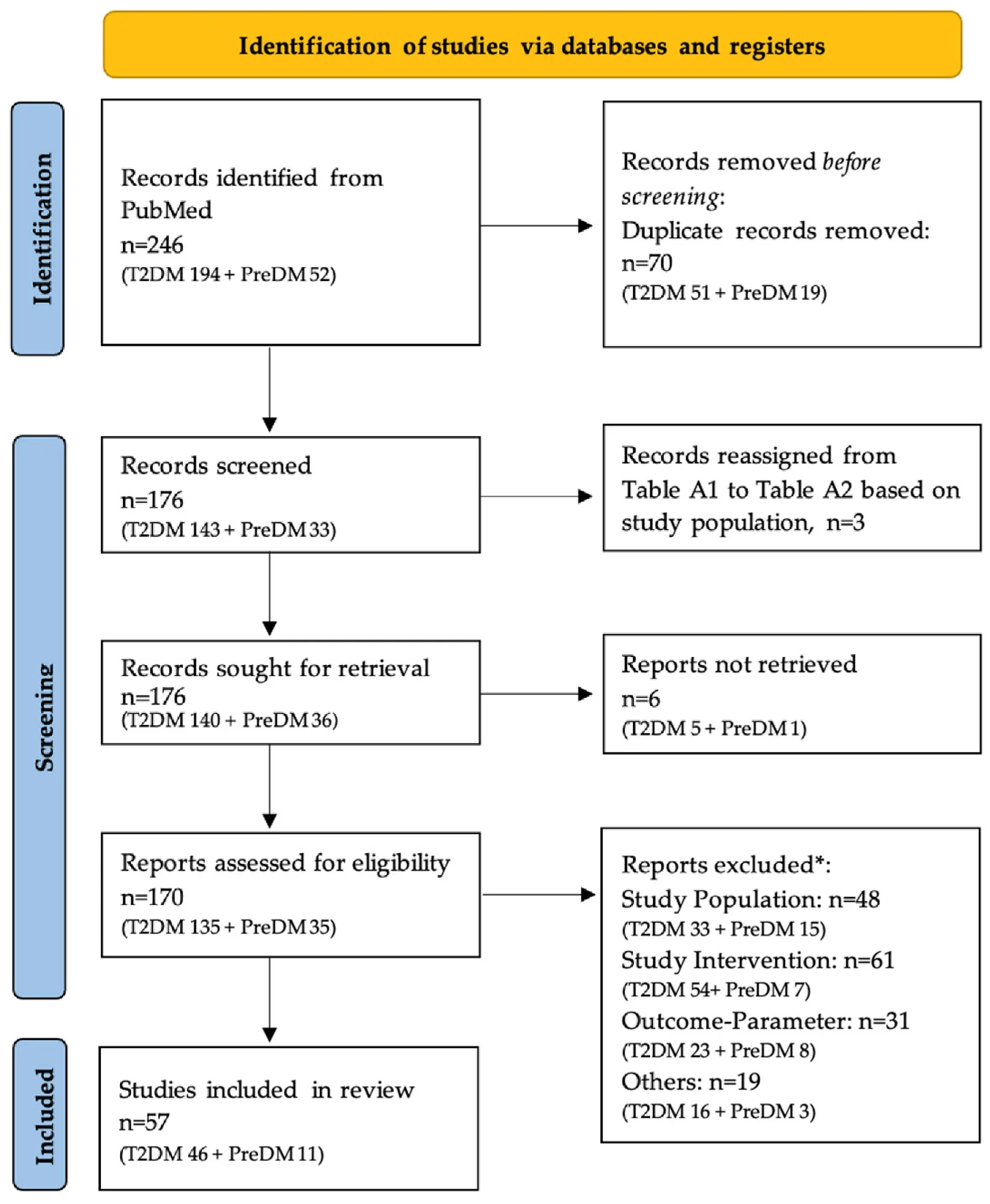
PRISMA flow diagram of article screening and inclusion. * Some reports were excluded, due to more than one exclusion criteria; n, Number; PreDM, Prediabetes; T2DM, Type 2 Diabetes mellitus.

**Table 1:** Diagnosis Criteria for Prediabetes and Diabetes mellitus.

	Fasting Plasma Glucose (FPG)	Hemoglobin A1c (HbA1c)	Oral Glucose Tolerance Test (2 h-OGTT)	Random Glucose
Normal	<100	<5.7	<140	-
Pre-diabetes	100–125	5.7–6.4	140–199	-
Diabetes mellitus	≥126	≥6.5	≥200	≥200

FPG, Fasting Plasma Glucose (mg/dL); HbA1c, hemoglobin A1c (%); OGTT, Oral Glucose Tolerance Test (mg/dL), Random Glucose (mg/dL).

## Data Availability

No new data were created or analyzed in this study and, therefore, data sharing is not applicable.

## Abbreviations

The following abbreviations are used in this manuscript:

ANOVA	Analysis of Variance
ATP III	Adult Treatment Panel III
AUC	Area Under the Curve
BLG	β-Lactoglobulin
CDY	Calcium + Vitamin D-fortified Yogurt Drink
CGM	Continuous Glucose Monitoring
CLA	Conjugated Linoleic Acids
cMax	Maximum Concentration
DY	Vitamin D-fortified Yoghurt Drink
DPP-4	Dipeptidyl Peptidase-4
FBG	Fasting Blood Glucose
FFM	Fat-Free Milk
FFY	Full-Fat Yogurt
FPG	Fasting Plasma Glucose
FSG	Fasting Serum Glucose
FY	Vitamin D-Fortified Low-Fat Yogurt
GIP	Glucose-dependent Insulinotropic Polypeptide
GMI	Glucose Management Indicator
GLP-1	Glucagon-like Peptide-1
g	grams
HbA1c	Hemoglobin A1c
HLA	Human Leukocyte Antigen
HOMA-β	Homeostasis Model Assessment of Beta-Cell Function
HOMA-IR	Homeostasis Model Assessment of Insulin Resistance
iAUC	Incremental Area Under The Curve
IGT	Impaired Glucose Tolerance
IFG	Impaired Fasting Glucose
IR	Insulin Resistance
ISF	Interstitial Fluid
IU	International Units
LFM	Low-Fat Milk
MCT	Medium-Chain Triglyceride
MD19	Maltodextrin 19
MFGM	Milk Fat Globule Membrane
mg	Milligrams
mL	Milliliters
MPCIR	Milk Powder Co-Supplemented With Inulin And Resistant Dextrin
MUFA	Monounsaturated Fatty Acid
NFM	Non-Fat Milk
NFY	Non-Fat Yogurt
OGTT	Oral Glucose Tolerance Test
OLL2712	Lactiplantibacillus Plantarum OLL2712
OWIST	Oral Whey Protein Stimulation Test
PY	Plain Yogurt Drink or Plain Low-Fat Yogurt Control Group
PRISMA	Preferred Reporting Items for Systematic Reviews and Meta-Analyses
PRT	Progressive Resistance Training
PUFA	Polyunsaturated Fatty Acids
RCTs	Randomized Controlled Trials
SC	Sodium Caseinate
SFA	Saturated Fatty Acids
SSC	Sugar-Sweetened Coffee
T2DM	Type 2 Diabetes mellitus
T1DM	Type 1 Diabetes mellitus
WM	Whole Milk
WPI	Whey Protein Isolate

## Appendix A

**Table A1:** Identified literature in adults with type 2 diabetes grouped by intervention.

Author and Year	Sample Size and Population	Dietary Exposure/Intervention	Follow-Up	Outcomes	Main Metabolic Findings
Whey Protein Preloads and Acute Administration					
Smith et al.; 2023 [74]	n = 18 T2DM adults	15 g whey protein 10 min vs. Placebo (0 g whey protein) 10 min before breakfast with mixed-meal tolerance tests (MMTTs)	2 × 240 min post-prandial	PPG iAUC, β-cell function, glucacon, insulin, c-peptide, incretin hormones	With whey protein: PPG iAUC: −16% (*p* < 0.05); peak glucose: −1.5 mmol/L (*p* < 0.05); β-cell function: +40% (*p* = 0.001). Insulin clearance postprandial: −22% (*p* ≤ 0.0001); glucagon and GLP-1 iAUC: 5.9-fold increase (*p* < 0.0001); insulin iAUC: 1.5-fold increase (*p* < 0.001.)
Smith et al.; 2022 [101]	n = 18 T2DM adults	15 g whey protein shot vs. nutrient-depleted placebo beverage 10 min before each meal	7 days (free-living)	blinded CGM data; time in range (TIR 3.9–10 mmol/L), time above range (TAR > 10 mmol/L), time below range (TBR < 3.9 mmol/L), mean glucose	TIR: +9% (~2 h/day) (*p* < 0.05); TAR: −8% (*p* < 0.05); mean glucose: −0.6 mmol/L (*p* < 0.05). Nocturnal glycemic control unaffected.
Ma et al.; 2015 [99]	n = 7 T2DM adults	Chocolate-flavored preload (100 mL water with either 25 g whey protein isolate or 25 g chocolate-flavored ‘diet’ sauce as a placebo) 30 min before each of the three main meals for 4 weeks Standardized study meal: mashed potato meal 65 g + 20 g glucose in 250 mL water	2 × 4 weeks; 2 weeks’ washout	PPG, peak glucose, fructosamine, gastric emptying	The capacity of the 25 g whey preload to effectively slow gastric emptying and significantly reduce postprandial glycemia persisted even after 4 weeks of regular exposure. PPG (*p* < 0.05) and peak glucose lower (12.5 ± 0.8 mmol/L vs. 13.8 ± 0.5 mmol/L, *p* = 0.005) and gastric emptying slower (*p* < 0.0005) after whey protein first intake; after 4 weeks blood glucose lower (*p* < 0.05), but peak glucose not significantly different (12.7 ± 0.7 mmol/L vs. 13.3 ± 0.7 mmol/L, *p* = 0.10), gastric emptying slower (*p* < 0.0005); Fructosamine & HbA1c: no significant difference.
Jakubowicz et al.; 2014 [77]	n = 15 T2DM adults	(1) Pre-meal drink (preload) containing 50 g whey protein concentrate dissolved in 250 mL water, administered 30 min before a high-glycemic-index breakfast (2) Control: 250 mL water (placebo) administered 30 min before the same breakfast	acute, 180 min postprandial	PPG, insulin, c-peptide, incretine hormones (GLP-1), DPP4-activity (AUC)	Whey preload: reduced the postprandial glucose AUC by 28% over 180 min compared with the placebo (*p* = 0.001). This glucose-lowering effect was accompanied by significantly higher insulin (+105%, *p* = 0.003), c-peptide (+43%, *p* = 0.0047) and intact GLP-1 (+298%, *p* = 0.0001) responses (increased AUCs).
Bjørnshave et al.; 2018 [109]	n = 12 T2DM and n = 12 matched controls (no DM2)	20 g Whey pre-meal vs. water 15 min before fat-rich meal (supplemented with 20 g WP in case of water pre-meal) +1.5 g paracetamol (3 × 500 mg)	acute, 360 min post-prandial; 1 week washout	FPG, PPG, insulin, glucagon, incretin hormones	WP increased early insulin (266.9 vs 179.9 pmol/L, *p* < 0.05) and glucagon (153.2 vs. 118.5 pg/mL, *p* < 0.05) at 15 min; no effect on TG or glucose responses.
Wildová et al.; 2017 [110]	n = 16 T2DM adults	OWIST: “Oral Whey Insulin Stimulation Test“ 75 g whey protein concentrate (dissolved in water) vs. OGTT: 75 g oral glucose load (control).	acute post-prandial 180 min	Serum C-peptide, plasma glucose levels	OWIST stimulated C-peptide secretion (peak 2.91 nmol/L at 90 min) did not cause an increase in glycemia (*p* < 0.01 compared to OGTT). C-peptide iAUC was 32% lower and peak levels 22% lower after whey, compared to the glucose load (OGTT).
Whey Protein—Formulations and Comparisons with Other Proteins					
King et al.; 2018 [76]	n = 11 T2DM men	(1) 15 g intact whey (IW) protein concentrate vs. (2) 15 g hydrolyzed whey (HW) protein vs. (3) placebo: flavored water immediately followed by mixed-nutrient breakfast and lunch meals (3 h in between)	acute postprandial (180 min) and CGM 24 h; 3 days, washout 7 days	Primary: PPG AUC Secondary: insulin, incretin/appetite hormones (GLP1-1, GIP, leptin, PYY), interstitial glucose with CGM; amino acids, satiety	IW: reduced total PPG AUC by 13 ± 3% after breakfast vs. control (*p* < 0.05); HW attenuated early PG after breakfast vs. control (*p* < 0.05); glycemia improved post lunch after IW vs. control (*p* < 0.05). IW and HW increased insulin (*p* < 0.05).
Smedegaard et al.; 2021 [102]	n = 16 T2DM adults	25 g β-lactoglobulin (BLG) vs. 25 g WPI taken 30 min before a 75 g oral glucose tolerance test (OGTT) or breakfast. 4 days with CGM after oGTT, four standardized meals; protein pre-meal 30 min before breakfast and dinner on 2 days and water as a control on days without pre-meal protein at home	180 min (acute oGTT) and 4 day of CGM at home; 1 to 6 week(s)’ washout	Insulin, Glucagon, Glucose, C-Peptide, iAUC, Coefficient of Variation (CV), mean glucose, amino acids, incretin hormones (interstitial fluid concentration, ISF)	OGTT: BLG increased insulin (+10%), glucagon (+20%), and glucose (+10%) significantly more than WPI (*p* < 0.05). CGM: Both proteins reduced ISF-glucose by ~2 mM and lowered CV by 10–15% vs. water (*p* < 0.05). WPI was more potent than BLG, lowering glucose by an additional 0.5 mM from 120 min onwards (*p* < 0.05).
Mortensen et al.; 2012 [108]	n = 12 T2DM adults	4 fat-rich meals (containing 100 g butter + 45 g carbohydrates) combined with 4 protein fractions: (1) Iso-meal: WPI (2) Hydro-meal: whey protein hydrolysate (3) Lac-meal: lactalbumin-rich whey protein (4) CGMP-meal: Casein glycomacropeptide-rich whey protein	acute, 480 min postprandial	Postprandial glucose, insulin, glucagon, lipids, incretin hormones (GIP, GLP-1), FFA	Iso and hydro-meal: caused higher insulin response compared to Lac and CGMP meal (*p* < 0.001); iAUC for the glucose response significantly higher up to 120 min after the hydro-meal than after the CGMP-meal (177 ± 109 vs. 121 ± 79, mean iAUC 120 min ± s.d., respectively; *p* = 0.035) iAUC of GIP significantly lower for the hydro-meal than for the Iso-meal, up to 60 min (*p* = 0.019) and 120 min (*p* = 0.009). GLP-1 response (iAUC up to 30 min) was significantly higher for the hydro-meal compared with the CGMP-meal (*p* = 0.045). No diff in triglycerides.
Derosa et al.; 2020 [100]	n = 120 T2DM, white (end: n = 59 + 58)	Daily, before breakfast: WPI (≥92.5%, native cysteine 2.7%, lactoferrin ≥0.7%) vs. placebo (5 g caseins) in 100 mL water	3 months	FPG, HbA1c, insulin, HOMA-IR, lipids, BMI, oxidation + inflammation markers	WPI group showed reduced FPG (−8.22%, *p* < 0.05), no other significant results for glycemia; improved oxidation/inflammation markers (*p* < 0.05).
Tessari et al.; 2007 [78]	n = 12 T2DM adults	Acute administration of a medium-calorie, high-protein mixed meal (6 kcal/kg body weight; approx. 31% carbs, 17% lipids, 52% proteins as total calories) on 3 separate occasions. Protein sources: (1) WHEY: Fast-absorbing sweet whey protein isolate (2) CASEIN: Slow-absorbing micellar casein (3) FREE AA: A free L-amino acid mixture matching the exact amino acid composition of micellar casein	acute, 180 min postprandial; 2 weeks’ washout	PPG, insulin, C-peptide, glucagon, incretin hormones (GLP-1, GIP), amino acids, lipids	Consuming WHEY stimulated a 12–40% greater insulin, C-peptide, and proinsulin response (AUC) compared to CASEIN (*p* < 0.02 or less). Postprandial glucose concentrations were nearly identical between the WHEY and CASEIN visits. However, the FREE AA meal led to significantly lower glucose compared to CASEIN (Cmax *p* = 0.002 and AUC_0-180_ *p* = 0.015); total GIP AUC was significantly lower after FREE AA compared with both WHEY (−40%) and CASEIN (−30%) (*p* < 0.01).
Whey Protein—Breakfast and Meal-Replacement Formulations					
Jakubowicz et al.; 2017 [73]	n = 48 T2DM adults	Breakfast different in 3 groups; lunch and dinner similar, diets isocaloric (1) whey protein breakfast diet (WBdiet): 42 g total protein, 28 g are whey; (2) protein breakfast diet (PBdiet): 42 g protein mainly from eggs (7 g), tuna (20 g), soy (7 g); (3) high-carbohydrate breakfast diet (CBdiet) 17g soy protein	12 weeks	HbA1c, c-peptide, FPG, AUC postprandial glucose, insulin, body weight, satiety, incretin and appetite hormones	HbA1c reduced after WBdiet by (−0.89%), PBdient (−0.6%), CBdiet (−0.36%; *p* < 0.001 for all); postprandial glucose reduced by PBdiet (12%) and WBdiet (19%, *p* < 0.001 for both) vs. CBdiet; WBdiet: greater postprandial overall AUC for insulin, C-peptide, GLP1 (*p* < 0.001); mean FPG: decreased in all 3 groups (greatest reduction in WBdiet, −11%).
Sridonpai et al.; 2021 [104]	n = 15 T2DM adults	Whey protein-based multi-ingredient nutritional drink (WD) vs. boiled white rice with chicken (BC)	240 min; 2 test meals, 1 week washout	Glucose iAUC, insulin iAUC, GLP-1	iAUC glucose significantly lower with WD; active GLP-1 iAUC significantly higher (*p* < 0.001).
Whey Protein—Supplementation with Exercise					
Soares et al.; 2023 [83]	n = 26 T2DM men	20 g whey protein isolate vs. maltodextrin isocaloric drink; both groups with resistance training (RT) 2 times/week	12 weeks	Muscle strength, body composition, glycemic control (Fructosamine, HbA1c, Glucose, Insulin, HOMA-IR), renal function	No significant differences between groups for performance or glycemic control.
Miller et al.; 2021 [82]	n = 167 T2DM	PRT + ProD: Progressive resistance training (2–3 d/wk) plus Whey Protein (20 g morning + 20 g post-exercise) and Vitamin D (2000 IU/d) vs. PRT: exercise only (no supplement, no placebo)	24 weeks	Primary: HbA1c, HOMA2-IR Secondary: FPG, insulin, c-peptide, body composition, lipid, blood pressure	No significant additive benefit of supplementation on HbA1c (PRT + ProD −0.10% vs. PRT −0.17%, *p* = 0.322) or HOMA2-IR (*p* = 0.370) after 24 weeks. FPG improved significantly more in the PRT-only group (net difference 0.6 mmol/L, *p* = 0.018).
Gaffney et al., 2018 [84]	n = 24 T2DM men	High-intensity mixed-mode interval training (MMIT) plus post-exercise: (1) 40 g/day whey protein (20 g before/after) vs. (2) isocaloric carbohydrate placebo	10 weeks	Primary: glucose disposal rate (GDR) via modified hyperinsulinemic-euglycemic clamp. Secondary: fasting blood glucose (FBG), insulin, HOMA-IR	MMIT improved insulin sensitivity (GDR) in both groups (+25–27%, *p* < 0.05). Whey protein did not further enhance glycemic control or GDR, compared to exercise with placebo.
Pabla et al.; 2025 [103]	n = 21 T2DM adults (n = 18 MCT, n = 19 MCT + WPI, n = 20 control)	(1) MCT: 15 g medium-chain triglycerides (MCTs) before breakfast and water before lunch and dinner; (2) MCT + WPI: 15 g MCT before breakfast + 10 g whey protein isolate (WPI) before lunch and dinner; (3) Control: water before each meal	3 × 24 h	Primary: diurnal glucose iAUC (08:00–23:00) by CGM Secondary: postprandial plasma glucose (PPG) after breakfast/lunch changes, insulin, c-peptide, proinsulin, glucagon, incretin hormones (GIP, Glucose-dependent Insulinotropic Polypeptide; GLP1, Glucagon-like Peptide 1; PYY, Peptide YY); indices of 24 h glycaemia; β-hydroxybutyrate (b-HBT)	No differences in diurnal and 24 h iAUC. MCT and MCT + WPI: lower 24 h glycemic variability (16.8%, *p* = 0.033 and 16.1%, *p* = 0.0004); PPG iAUC 17% lower after MCT and MCT +WPI breakfast; MCT + WPI −20% glucose 8:30–17:30 (*p* = 0.002); GIP, PYY and b-HBT higher in MCT and MCT + WPI. Lower appetite after MCT + WPI breakfast and lunch.
Wu et al.; 2016 [75]	n = 22 T2DM men	(1) 50mg vildagliptin (VILD) (2) or placebo (PLBO) (1) + (2) evening before and morning of each study day; morning 60 min after VILD/PLBO (3) preload drink with 25 g whey protein (WHEY); or (4) control flavoring (CTRL) after another 30 min mashed potato meal	acute post-prandial, 240 min	PPG, insulin, incretin hormones (GLP-1, GIP), gastric emptying	PLBO/WHEY: improved postprandial glycemia, increased insulin, glucagon and incretins (*p* < 0.05) VILD/WHEY: enhanced the glucose-lowering efficacy, improved the postprandial insulin and incretin response compared to either treatment alone (*p* < 0.05).
Other Protein Comparisons—Milk Versus Soy Protein and Casein or Whey Soy Supplementation					
Gobert et al.; 2010 [81]	n = 29 T2DM adults	(1) MPI: 40 g Milk Isolate vs. (2) SPI: 40 g Soya Isolate 2 × 29 g packets/d daily total of 837 kJ (8–9 g carbs, 40 g protein, 88 mg (SPI) or 0 mg (MPI) isoflavones, 1 g fat, 0–10 mg cholesterol and 1400–1600 mg Ca)	2 × 57 days; 4 weeks’ washout	FPG, fasting insulin, PPG (maximum concentration (Cmax), time to Cmax (Tmax), AUC), HbA1c, HOMA-IR, HOMA-B, QUICKI, Matsuda index	OGTT, indices for insulin resistance/sensitivity, HbA1c, FPG and fasting insulin: no difference between groups.
Ang et al.; 2012 [111]	n = 30 T2DM adults	(1) ISO + C: drink containing 50 g isomaltulose combined with 21 g casein (2) ISO + WS: drink containing 50 g isomaltulose combined with 21 g whey/soy mixture (1:1 ratio) (3) Control (ISO): drink containing 50 g isomaltulose alone	acute, 240 min postprandial; 3 days’ washout	PPG, postprandial insulin response, insulin action/sensitivity index (SI)	No significant differences in glucose responses were observed between the drinks. Compared to ISO, the ISO + C and ISO + WS resulted in a significantly higher postprandial insulin response (~190–270%, *p* < 0.001), but significantly lower insulin action/sensitivity (*p* < 0.01 and *p* = 0.005). Plasma insulin levels increased more significantly (*p* < 0.001) after ingestion of ISO + WS compared to ISO + C, and were positively correlated with total amino acid levels (*p* < 0.001). Insulin sensitivity was significantly lower following ISO + WS compared to ISO + C (*p* = 0.003); dairy protein stimulated insulin secretion, but did not improve the overall glucose response.
Dairy Diets—Low-Fat Versus High-Fat and Dairy Versus Non-Dairy					
Mitri et al.; 2020 [79]	n = 111 T2DM adults (38–36–37 in intention to treat)	(1) control group: maintained baseline dairy intake (<3x/d) vs. (2) low-fat (LF, <2%) dairy (≥3 servings/d) vs. (3) high-fat (HF, ≥2%) dairy (≥3 servings/d)	24 weeks	Primary: HbA1c, secondary: FPG, HOMA-IR, body weight, BMI, lipids	No significant differences in HbA1c, FPG, HOMA-IR, lipids, body weight or BP between groups after 24 weeks.
Tsameret et al.; 2026 [70]	n = 25 T2DM adults (n = 19 completed/analyzed)	Dairy-protein sources (YesMilk) vs. isoenergetic non-dairy)	2 × 4 weeks; 3–4 weeks’ washout	Fasting glucose, time in range (TIR)	Fasting glucose: −1.7 mmol/L; TIR: +9%; Glucose Management Indicator: −0.7% vs. baseline (all *p* < 0.05).
Milk—Comparisons with Plant-Based Milk and Other Beverages					
Dhaver et al.; 2025 [106]	n = 22 T2DM + overweight or obesity	40 g oatmeal served with 360 mL almond milk (ALM), 246 mL carbohydrate-matched 2% milk (MLKCRB) or 182 mL calorie-matched 2% milch (MLKCAL) on separate days	4 h post-prandial; study duration 6 weeks	Primary: glucose incremental area under the curve 240 min (iAUC); secondary: postprandial insulin, glucagon, free fatty acids, triglycerides, leptin, gastrointestinal hormones	No difference in glucose iAUC; higher insulin and glucagon iAUC with carbohydrate-matched 2% milk vs. almond milk (*p* < 0.05); no other effects on primary or secondary endpoints.
Miraghajani et al.; 2013 [80]	n = 29 T2DM (nephropathy)	Soy milk vs. cow’s milk diet	2 × 4 weeks; 2 weeks’ washout	FPG, serum insulin, Hba1c, BP, lipids, markers of kidney function	No significant results between cow´s milk and soy milk for glycemia. Soy milk significantly reduced systolic BP vs. cow’s milk (percent change: −4.5 vs. +5.8%, *p* = 0.03).
Milton et al.; 2007 [133]	n = 6 T2DM; n = 6 healthy with overweight (control)	300 mL skimmed milk (low glycemic index (GI) = 32) vs. 300 mL Ovaltine (high GI = 105) vs. 300 mL water preload	acute, 200 min postprandial	PPG iAUC, insulin iAUC, GLP1 iAUC, gastric emptying rate	Post-Preload Glycemia (iAUC_0–20): in both groups, Ovaltine produced the greatest glucose rise, followed by milk, and water (*p* < 0.001). In the T2DM group, milk caused a small rise compared to water (*p* = 0.03). Post-Preload Insulin (iAUC_0–20): no significant insulin difference. Insulin-to-glucose ratio (relative to water as 1.0) on the milk visit was 6.82 for control and 1.78 for T2DM; GLP-1 Response (iAUC_0–20): control group: the initial GLP-1 response was higher after both milk and Ovaltine compared with water (*p* = 0.041). T2DM: no difference in GLP1. Total Postprandial Response (iAUC_0–200): no significant differences were observed between the three preload days for total 200 min glucose, insulin, or GLP-1 responses in either group.
Dairy Desserts—Ice Cream Versus a Soy-Based Dessert					
Bukar et al.; 1990 [107]	n = 12 TDM adults	3 separate test meals (1) Glucose standard: 50 g glucose powder dissolved in water (2) Ice Cream (IC): 50 g of dairy-based sucrose-sweetened chocolate ice cream (containing milk fat, non-fat milk solids, sucrose, lactose, corn syrup) (3) Tofu Frozen Dessert (TFD): 50 g of cholesterol-free tofu-based frozen dessert (water, soybean curd/tofu, high-fructose corn syrup, corn oil) dairy-based ice cream vs. tofu dessert	acute, 180 min post-prandial; 2 days’ washout	PPG, insulin, peak glucose, peak insulin, glucose AUC, insulin AUC, GI	Peak glucose occurred at 120 min for both desserts; peak glucose response was significantly lower after IC compared with the TFD (190 ± 11 vs. 222 ± 13 mg/dL, *p* < 0.01) Postprandial glucose iAUC: smaller for IC than for TFD (7194 vs. 12,302 mg/dlxmin, *p* < 0.05); FPG and peak insulin response not different between the IC and TFD visits.
Enriched Dairy Products—Calcium-Rich Milk					
Costa et al.; 2020 [71]	n = 14 T2DM adults	(1) Drink with fat-free milk powder + water with 700 mg Calcium (DAIR) vs. (2) Water, whey protein, vitamins D, C, A, Fe and sodium chloride, 0 mg Calcium (CONT) (1) + (2): energy-restricted diet (−500 kilocalories (kcal)/d; 800 mg/d Calcium), nutritional education	90 days; 60 days’ washout	HbA1c, fasting serum glucose (FSG), insulin, HOMA-IR, food intake, body composition, lipids, other	HbA1c (7.1 à 6.4, *p* < 0.05 in DAIR; −0.7 difference, *p* < 0.025 inter-groups) and FSG (inter-groups, *p* < 0.025) reduced significantly in DAIR vs. CONT. HOMA-IR: no difference.
Gomes et al.; 2019 [85]	n = 14 T2DM adults	HC: 1200 mg/d Ca (high-Ca fat-free milk shake with 700 mg Ca) vs. LC: 525 mg/d Ca (low-Ca shake control with 6.4 mg Ca); both groups: breakfast shake, energy-restricted diet (800 mg Ca/d)	2 × 12 weeks; 8 weeks’ washout	HbA1c, HOMA2-%B, HOMA2-IR, fructosamine, FPG, Body fat, BMI, other	HC significantly reduced HbA1c (7.1% to 6.4%) and improved HOMA2-%B compared to baseline (*p* < 0.05) after 12 weeks. HC: FPG (*p* = 0.02), HbA1c (*p* = 0.001) decreased, HOMA2-%B (*p* = 0.016) increased after 12 weeks, compared with LC. Note: Baseline HbA1c higher in HC at baseline.
Gomes et al.; 2018 [134]	n = 14 T2DM adults	HC (High Calcium): Breakfast shake with 700 mg Ca from fat-free milk powder plus energy-restricted diet (−500 kcal/d) vs. LC (Low Calcium): Iso-caloric/iso-proteic shake (no milk) plus energy-restricted diet	2 × 12 weeks; 8 weeks’ washout	Metabolic syndrome components, FPG, lipids, BP, other	Fasting glucose remained stable in HC but increased in the LC group (from 6.9 to 8.3 mmol/L, *p* < 0.05).
Fortified Versus Unfortified Dairy—Vitamin D in Milk					
Salehi et al.; 2018 [91]	n = 101 T2DM adults	(1) unfortified low-fat milk (250 mL/day) vs. (2) low-fat milk fortified (250 mL/day + 1000 IU vitamin D3) & additional 2 servings of non-fortified milk and dairy products	9 weeks	FPG, insulin, HbA1c, lipids, Vit D, Ca, body composition, BMI, blood pressure (BP), other	Fortified milk improved Vitamin D status but did not affect metabolic markers vs. plain milk. HbA1c decreased in both groups, but more in plain milk group (*p* = 0.02).
Fortified Versus Unfortified Dairy—Vitamin D with or without Added Calcium in Yogurt and Yogurt Drinks					
Nikooyeh et al.; 2021 [87]	n = 75 T2DM adults	(1) DY: Vitamin D-fortified yogurt (1000 IU Vitamin D, 300 mg Calcium) (2) CDY: Calcium + Vitamin D-fortified yoghurt (1000 IU Vitamin D, 500 mg Calcium) (3) PY: plain yoghurt (no Vitamin D, 300 mg Calcium)	12 weeks	Adiponectin, Sirtuins HbA1c, body mass index (BMI), total body fat mass (FM)	Significant decrease in HbA1c, BMI and FM compared to baseline in DY and CDY group. HbA1c increased with PY. Significant increment in serum adiponectin in both DY and CDY groups (*p* < 0.001). SIRtuin1 and 6 significant within-group increment in CDY group (*p* = 0.003; *p* = 0.001)
Nikooyeh et al.; 2021 [135]	n = 75 T2DM adults	(1) DY: Vitamin D-fortified yogurt (1000 IU Vitamin D) (2) CDY: Calcium + Vitamin D-fortified yoghurt (1000 IU Vitamin D, 500 mg Calcium) (3) PY: plain yoghurt (no Vitamin D)	12 weeks	Metabolic rate (RMR), TSH, HOMA-IR	HOMA-IR improved in correlation with rising 25-OH Vitamin D levels (*p* = 0.001). Mean RMR increased significantly in both DY and CDY groups (*p* < 0.001).
Jafari et al.; 2016 [88]	n = 59 T2DM postmenopausal women	(1) FY: 100 g/day of vitamin D-fortified low-fat yogurt (2000 IU vitamin D) vs. (2) PY = control: 100 g/day of plain low-fat yogurt Equivalent amounts of dairy products in both groups	12 weeks	FPG, fasting insulin, HOMA-IR, HOMA-B, QUICKI, HbA1c, lipids, other	FY: improvements in serum fasting insulin, HOMA-IR, HOMA-B, and QUICKI compared to baseline, while these markers worsened in PY group. Between groups: FY group better final values for all glycemic indexes (including FPG, *p* = 0.026) except for HbA1c (*p* = 0.227).
Shab-Bidar et al.; 2015 [136]	n = 60 T2DM adults	(1) FD: 500 mL/d (2 × 250 mL bottles) of Vitamin D-fortified yoghurt drink (doogh) with 1000 IU vitamin D3/d and 300 mg Calcium) vs. (2) PD: 500 mL/d of plain yoghurt drink (300 mg Calcium, no Vitamin D)	12 weeks	FPG, HbA1c, fasting insulin, QUICKI, central adiposity measures, other	FD vs. PD: FD improvements in QUICKI (*p* < 0.001), but not in HbA1c, FPG. Metabolic response to the FD was dependent on the genetics (Vitamin D Receptor (VDR) Caudal-Type Homeobox Protein 2 Cdx-2 polymorphism).
Shab-Bidar S et al.; 2011 [89]/Hajimo-hammadi et al.; 2017 [90]	2011: n = 100 T2DM adults 2017: n = 97	(1) Intervention = FD/FYD (fortified yoghurt = doogh): 2 × 250 mL/d of yogurt drink fortified with 500 IU Vitamin D3 and 170 mg Calcium per bottle vs. (2) Control = PD/PYD (plain yoghurt = doogh): 2 × 250 mL/d with 170 mg Calcium but no Vitamin D	12 weeks	HbA1c, FSG, insulin, QUICKI, lipids, endothelian biomarkers (endothelin-1, matrix metalloproteinase), appetite hormones (leptin, ghrelin); other	**FSG:** decreased in FYD (10.5 ± 3.1 to 9.0 ± 2.8 mmol/L, *p* = 0.001); **Insulin:** decreased in FYD (*p* = 0.002) and increased in PYD (*p* = 0.01; between-group *p* = 0.001, ANCOVA *p* < 0.001); QUICKI: increased in FYD (0.29 to 0.30, *p* = 0.001) but decreased in PYD (0.28 to 0.27, *p* = 0.041; between-group *p* = 0.001, ANCOVA *p* < 0.001); HbA1c: decreased in FYD (8.7% to 7.8%, *p* = 0.001); difference in changes not significant after ANCOVA adjustment (*p* = 0.10).
Nikooyeh et al.; 2011 [86]	n = 90 T2DM adults	2 bottles per day with (1) DY: vitamin D-fortified doogh (150 mg calcium and 500 IU vitamin D/250 mL) (2) CDY: calcium- and vitamin D-fortified doogh (250 mg calcium and 500 IU vitamin D/250 mL) (3) PY: plain doogh (150 mg calcium and no detectable vitamin D/250 mL)	12 weeks	FPG, HbA1c, fasting insulin, HOMA-IR, lipids, other	FPG: decreased in DY (−12.9 mg/dL, *p* = 0.015) and CDY (−9.6 mg/dL, *p* = 0.035) compared to the PY group (+16.3 mg/dL, *p* < 0.001); HbA1c: increased in PY group (+1.2%, *p* < 0.001), optimized/stabilized in DY (−0.4%, *p* < 0.001) and CDY (−0.4%, *p* < 0.001), with between-group differences favoring DY and CDY (*p* < 0.001 for both vs. PY); HOMA-IR: worsened in the PY group (+2.1), but improved in DY (−0.6, *p* = 0.001) and CDY (−0.6, *p* < 0.001), with significant between-group differences vs. control (*p* ≤ 0.001).
Fortified Versus Conventional Dairy—B Vitamins in Yogurt					
Yanni et al.; 2019 [92]	n = 33 T2DM adults (n = 28 analyzed)	VY: non-fat yoghurt enriched with vitamins B1 (0.8 mg), B5 (4 mg) and B6 (1.2 mg) vs. CY: conventional yoghurt with higher protein (19.2 g vs. 9.8 g per 200 g) and lower carbohydrates (8.4 g vs. 15.4 g per 200 g) compared to VY 2 × 200 g/d	12 weeks 2 weeks’ run-in	HbA1c, FPG, fasting insulin, body weight, BMI, IL-6, other	VY: HbA1c not significantly reduced (6.2 vs 6.4%, *p* = 0.08). FPG did not change. VY reduced weight/BMI compared to baseline (*p* < 0.05); no between-group results for glycemia reported.
Supplemented Versus Plain Milk Powder—Inulin and Resistant Dextrin					
Cai et al.; 2018 [72]	n = 99 elderly T2DM adults	45 g milk powder + inulin + resistant dextrin (MPCIR) vs. 45 g Placebo milk powder Both: 30 g before breakfast in 160 mL water and 15 g in 80 mL water before dinner	12 weeks	Primary: FPG, secondary: 2 h PPG, HbA1c, c-peptide, insulin, BP, HOMA-IR	MPCIR decreased fasting (−0.96 mmol/L) and 2 h postprandial plasma glucose (−1.47 mmol/L), HbA1c (−0.38%) and HOMA-IR (−0.65, *p* = 0.002) of the MPCIR group (*p* < 0.001 for all others) are decreased. 2 h-pp insulin was increased (*p* = 0.016)
Milk with Versus without Plant Extracts—Mulberry Leaf and Corn Silk					
Sun et al.; 2025 [105]	n = 28 T2DM	Whole-grain bread 50 g plus: (1) 440 mL water; (2) water + Mulberry/Corn Silk Extract (MCE) (3) 440 mL lactose-hydrolyzed milk (LHM); (4) LHM + MCE	2 h post-prandial; 7 days’ washout	Continuous glucose monitoring (CGM) data: iAUC, 1 h postprandial glucose (1 h PG), 2 h postprandial glucose (2 h PG), maximum postprandial glucose (max PG)	LMH vs. water: increased 2 h PG (8.3 vs. 7.4 mmol/L, *p* < 0.001) and the iAUC (1086 vs. 1023, *p* = 0.04); LHM-MCE vs. LMH: lower 1 h PG (−0.7 mmol/L, *p* = 0.04) and Max PG (−0.9 mmol/L, *p* = 0.014).
Probiotic and Fermented Dairy—Yogurt and Yogurt Drinks					
Mirjalili et al.; 2023 [93]	n = 72 T2DM adults	200 g/d probiotic yogurt (4.65 × 10^6^ colony-forming-unit (CFU)/gram (g) Lactobacillus acidophilus and Bifidobacterium lactis) vs. placebo (200 g/day (d) conventional yogurt)	12 weeks	FPG, HbA1c, Lipids	Significant reduction in HbA1c after adjusting (−0.76 vs. −0.15, *p* = 0.01), TC (−10.61 vs. −2.97, *p* = 0.02) and LDL-C (−8.62 vs. 0.02, *p* = 0.004) in probiotic group. No significant difference in FPG (but trend), TG and HDL-c.
Ejtahed et al.; 2012 [95]	n = 64 T2DM adults (n = 60 completed)	PY: consumed 300 g/day of probiotic yogurt containing *Lactobacillus acidophilus* La5 and *Bifidobacterium lactis* Bb12 vs. CY: control consumed 300 g/day of conventional yogurt	6 weeks	FPG, HbA1c, fasting insulin, antioxidative/oxidative stress markers	FPG: decreased in PY (from 8.06 to 7.36 mmol/L, *p* < 0.01 vs. baseline), CY unchanged compared to baseline. Significant between-group difference after 6 weeks (*p* < 0.05) HbA1c: within-group change in PY not significant (7.29 to 7.17%, *p* > 0.05); CY: HbA1c increased (from 6.87 to 7.17%, *p* < 0.01 vs. baseline); between-group difference in favor of PY after 6 weeks (*p* < 0.05) fasting Insulin: no change within or between either group (*p* > 0.05)
Hove et al.; 2015 [97]	n = 41 T2DM men	(1) 300 mL fermented milk (Cardi04 drinking yogurt), fermented with *Lactobacillus helveticus* vs. (2) Control: 300 mL placebo yogurt drink (prepared without bacteria, artificially acidified to match taste and macronutrients)	12 weeks	FPG, HbA1c, fasting insulin, C-peptide, HOMA-IR, lipid, inflammation markers, 24 h BP	The change in FPG differed significantly between the two groups, with a larger increase observed in the placebo group (*p* < 0.05). No significant differences between the fermented milk and placebo groups regarding changes in HbA1c, insulin, HOMA-IR. Note: The groups had significant baseline differences in fasting glucose and HbA1c, which were adjusted for in the analysis.
Probiotic and Fermented Dairy—Cow and Goat Milk					
Sato et al.; 2017 [96]	n = 70 T2DM (n = 68 for final analysis)	(1) Probiotic group: Lactobacillus casei strain Shirota-fermented milk vs. (2) Control group: no probiotics	16 weeks	HbA1c, FPG, fasting C-peptide, glycoalbumin, Gut microbiota, blood bacteria, other, lipids	HbA1c at 16 weeks in the control (+0.1%; *p* < 0.05) and probiotic (+0.2%; *p* < 0.01) groups were slightly increased compared with baseline. No significant differences between groups for HbA1c, FPG, glycoalbumin, c-peptide. Probiotic significantly lowered count of total blood bacteria (*p* < 0.05).
Tonucci et al.; 2017 [94]	n = 50 T2DM adults (n = 45 completed)	(1) Probiotic group: 120 g/d of probiotic fermented goat milk containing 10^9^ UFCs of L. acidophilus La-5 and 10^9^ UFCs of Bifidobacterium animalis subsp. lactis BB-12 (2) Control: 120 g/d of conventional fermented goat milk contained Streptococcus thermophilus TA-40 during the breakfast.	6 weeks	HbA1c, FPG, insulin, HOMA-IR, fructosamine, lipids, other	HbA1c improved in probiotic vs. control group (−0.65% vs. +0.31%, *p* = 0.02); reduction in fructosamine (−9.91mmol/L, *p* = 0.04) in probiotic group vs. baseline (but not inter-group difference).
Synbiotic Versus Probiotic and Placebo Formulations					
Zhang et al.; 2025 [98]	n = 120 T2DM	2g daily (after dinner): (1) Synbiotic mixture (MN-Gup + GOS) (2) Probiotic (MN-Gup) (3) Placebo (Maltodextrin)	12 weeks	Primary: Fasting Plasma Glucose (FPG); secondary: Hemoglobin A1c (HbA1c), insulin, Homeostatic Model Assessment of Insulin Resistance (HOMA-IR)	FPG (Between-group): MN-Gup-GOS lower than placebo (MD: −0.69 mmol/L [95% CI: −1.29, −0.10], *p* = 0.022). Within-group (MN-Gup-GOS): FPG −1.40 mmol/L (*p* < 0.001); HbA1c −0.31% (*p* = 0.034); insulin −0.83 mU/mL (*p* = 0.049); HOMA-IR −0.64 (*p* < 0.001). Between-group comparison: no significant difference for HbA1c, insulin, or HOMA-IR (*p* > 0.05).

1-h PG, 1-Hour_Postprandial Glucose (mmol/Lor mg/dL); 2-h PG, 2-Hour Postprandial Glucose (mmol/Lor mg/dL); BC, Boiled White Rice with Chicken; b-HBT, β-hydroxybutyrate; BLG, β-lactoglobulin; BMI, Body Mass Index (kg/m^2^); BP, Blood Pressure (mmHg; CBdiet, High-Carbohydrate Breakfast Diet; CDY, Calcium + Vitamin D-fortified Yoghurt; CFU, Colony-Forming-Unit; CGM, Continuous Glucose Monitoring; CGMP-meal, Casein Glycomacropeptide; Cmax, Maximum Concentration; CONT, Water, Whey protein, Vitamins (D, C, A, Fe and sodium chloride), no Calcium; CTRL, Control Group; CV, Coefficient of Variation; CY, Conventional Yoghurt with Higher Protein and Lower Carbohydrates; d, day; DAIR, Drink with Fat-Free Milk Powder + Water with Calcium; DY, Vitamin D-fortified Yogurt; FBG, Fasting Blood Glucose (mmol/L or mg/dL); FD/FYD, Fortified Yoghurt Drink; FM, Total Body Fat Mass; FPG, Fasting Plasma Glucose (mmol/L or mg/dL; FREE AA: Free L-Amino Acid Mixture; FSG, Fasting Serum Glucose (mmol/L or mg/dL); g, Gram; GDR, Glucose Disposal Rate; GI, Glycemic Index; GIP, Glucose-dependent Insulinotropic Polypeptide; GLP-1, Glucagon-like Peptide 1; HbA1c, Hemoglobin A1c (% or mmol/mol); HC, High-Calcium Fat-Free Milk Shake; HF, High-Fat; HOMA-IR, Homeostatic Model Assessment of Insulin Resistance; HW, Hydrolyzed Whey Protein; iAUC, Incremental Area Under the Curve (minutes); IC, Ice Cream; ISO + C: Drink Containing Isomaltulose Combined with Casein; ISO + WS: Drink Containing Isomaltulose Combined with Whey/Soy Mixture; IW, Intact Whey Protein Concentrate; ISF, Interstitial Fluid Concentration; Kcal, Kilocalories; Lac-meal, Lactalbumin-Rich Whey Protein; LC, Low-Calcium Shake Control; LF, Low-Fat; LMH, Lactose-Hydrolyzed Milk; Max.PG, Maximum Postprandial Glucose (mmol/Lor mg/dL); MCE, Mulberry/Corn-Silk Extract; MCTs, Medium-Chain Triglycerides; MMIT, High-Intensity Mixed-Mode Interval Training; MMTT, Mixed-Meal Tolerance Test; MPCIR, Milk Powder + Inulin + Resistant Dextrin; MPI, Milk Isolate OGTT, Oral Glucose Tolerance Test; n, number; PBdiet, Protein Breakfast Diet; PD/PYD, Plain Yoghurt Drink; PLBO, Placebo; PY, Plain Yoghurt or Probiotic Yogurt; PYY, Peptide YY; PPG, Postprandial Plasma Glucose (mmol/L or mg/dL); PRT, Progressive Resistance Training; PRT + ProD, Progressive Resistance Training plus Whey Protein and Vitamin D; RMR, Metabolic rate; RT, Resistance Training; SI, Insulin Sensitivity Index; SPI, Soya Isolate; TAR, Time Above Range; TBR, Time Below Range; TFD, Tofu Frozen Dessert; TIR, Time in Range (%); Tmax, Time to Cmax; T2DM, Diabetes Mellitus Type 2; WD, Whey Protein-based Multi-Ingredient Nutritional Drink; WHEY, Preload Drink with Whey Protein; VDR Cdx-2, Vitamin D Receptor Caudal-Type Homeobox Protein 2 Cdx-2 Polymorphism; VILD, Vildagliptin group; VY, Non-Fat Yoghurt Enriched with Vitamins B1, B5 and B6; WBdiet, Whey Protein Breakfast Diet; WPI, Whey Protein Isolate.

**Table A2:** Identified literature in adults with prediabetes grouped by intervention.

Author & Year	Sample Size & Population	Dietary Exposure/Intervention	Follow-Up	Outcomes	Main Metabolic Findings
Whey Protein—Preloads					
Bjørnshave et al., 2019 [116]	n = 20 Prediabetes/Metabolic syndrome	three pre-meals consisted of (1) control: 0g whey protein, 200 mL tap water preload (2) low-dose WP: 10 g whey protein isolate or (3) high-dose WP: 20 g of whey protein isolate followed by standardized, fat-rich breakfast meal (second meal)	360 min	Postprandial plasma glucose, insulin, glucagon, incretin hormones (GLP-1 and GIP), free fatty acids, lipids, paracetamol absorption kinetics	Postprandial Glycemia: dampening was observed only with the 20 g WP preload, with lower postprandial glucose response over the entire period compared to placebo (*p* = 0.0148), with reduction in glucose concentration at 30 min (*p* < 0.0001); Insulin Secretion (Dose-Dependent): WP preloads induced a highly significant dose-dependent increase in insulin responses (overall intervention effect *p* < 0.0001); Differences between all three doses: 0 g vs. 10 g (*p* = 0.027), 0 g vs. 20 g (*p* < 0.0001), and 10 g vs. 20 g (*p* = 0.0005); Glucagon Secretion (Dose-Dependent): Glucagon iAUC0–360 min showed a dose-dependent increase (overall *p* = 0.0022), with lower responses for 0 g vs. 10 g (*p* = 0.008) and 20 g (*p* = 0.001). The overall postprandial glucagon time-concentration curve differed across all three arms (*p* < 0.0001); Incretin Response (GLP-1 vs. GIP): 20 g WP preload increased GLP-1 concentrations compared to placebo (*p* = 0.0086), with elevations at the early 15 min peak (*p* = 0.001) and the late 120 min peak (*p* < 0.0001). No significant changes in the GIP response were observed between the arms. *Note: Inclusion criteria: HbA1c > 6.5%;* *Baseline characteristics: HbA1c 5.4%; FPG 104 mg/dL*
Milk Protein Hydrolysate—Preloads and Repeated Consumption					
Sartorius et al., 2020 [114]	n = 21 Prediabetes	Phase I: acute: single dose of (1) placebo: 1400 mg or 2800 mg of maltodextrin or (2) LD: low-dose 1400 mg bioactive peptides from milk protein hydrolysate = Pep2Dia® or (3) HD: high-dose 2800 mg (high dose) Pep2Dia® 15 min prior to a challenge meal high in carbohydrates Phase II: chronic: daily consumption of 1400 mg (low-dose) Pep2Dia® 15min before lunch for 6 weeks	Phase I: 180 min postprandial Phase II: long-term assessment after 6 weeks	Primary: postprandial glucose iAUC0–180 min Secondary: postprandial glucose maximum, increase (Cmax) Glucose AUC0-180 min, insulin iAUC0–180 min, Insulin Cmax, HbA1c, Matsuda index, HOMA-IR	Phase I: Primary Outcome (iAUC0–180 min glucose): acute ingestion of the low-dose (1400 mg) significantly reduced glucose iAUC compared to placebo (3441.1 vs. 4312.0 mg/dL × min, *p* = 0.0472). High-dose (2800 mg) did not differ significantly from placebo (non-linear dose–response); Glucose Excursions (Acute): low-dose significantly lowered ΔCmax above baseline (44.8 vs. 52.8 mg/dL, *p* = 0.0237), total AUC0–180 min (21,931 vs. 23,073 mg/dL × min, *p* = 0.0313), and absolute glucose Cmax (152.1 vs. 160.1 mg/dL, *p* = 0.0237); Insulin: no significant differences in postprandial insulin iAUC or cMax. Phase II (6 weeks): HbA1c & Glycemia: daily low-dose Pep2Dia® reduced HbA1c levels (5.69% compared to baseline 5.78%, *p* = 0.0244). A total of 52.4% (11 out of 21 subjects) reverted to normoglycemia (HbA1c < 5.7%) after 6 weeks; Final challenge meal at week 6, glucose iAUC remained significantly reduced vs. placebo (*p* = 0.037).
Dairy Proteins—Whey and Casein Comparisons with Carbohydrate Co–Ingestion					
Hoefle et al., 2015 [115]	n = 15 Prediabetes adults and n = 15 Healthy male controls (n = 30 overall)	3 liquid test drinks (300 mL volume): (1) Control (MD19): 50 g of maltodextrin19 alone or (2) WPI Drink: MD19 + combination with 5g of whey protein isolate (WPI) or (3) SC Drink: MD19 + combination with 50 g of sodium caseinate (SC)	3 × 240 min; 2 days’ washout)	Postprandial plasma glucose, plasma insulin, c-peptide, glucagon, incretin hormones (GLP-1, GIP), insulin index at 240 min, oro-cecal transit time, gastric emptying rate	Glycemic excursion: WPI & SC reduced postprandial glucose excursions compared with MD19 in prediabetic group over the 4 h period (*p* < 0.05); peak glucose reduced by 21% after both protein drinks compared to MD19 (*p* < 0.0001 for both), with no significant differences between WPI and SC; Insulin: WPI & SC increased postprandial insulin levels over 240 min in prediabetic subjects compared with MD19; peak insulin elevated by 96% after WPI and SC (*p* < 0.05); Insulin index at 240 min increased by WPI (23.3 ± 3.74) and SC (16.3 ± 1.64) compared to MD19 control (8.50 ± 0.62, *p* < 0.05) Glucagon: peak concentrations increased by 101% after both protein drinks compared to MD19 (*p* < 0.05).
McDonald et al., 2019 [120]	n = 23 Prediabetes	Four acute beverage trials: (1) GLU: 75 g glucose dissolved in 473 mL water (2) MILK: 75 g glucose dissolved in 473 mL non-fat dairy milk (16.5 g total milk protein) (3) WHEY: 5 g glucose plus 16.5 g whey protein isolate dissolved in 473 mL water (4) CASEIN: 75 g glucose plus 16.5 g sodium caseinate dissolved in 473 mL water	180 min	Postprandial plasma glucose and insulin, lipids, oxidative stress markers, other	Postprandial Glycemia: with MILK, WHEY, or CASEIN attenuation of postprandial glucose excursions compared to GLU (iAUC0–180 min: *p* < 0.001). Compared with GLU, glucose increases lower at 60–90 min and 180 min in MILK, at 30–120 min in WHEY, and at 60–120 min in CASEIN (*p* < 0.05); Insulin & Lipids: no significant differences between groups.
Dairy Diets—Increased Consumption of Predominantly Low-Fat Dairy					
Turner et al., 2015 [117]	n = 47; overweight (n = 27 normal glucose tolerance; n = 6 impaired fasting glucose; n = 14 impaired glucose tolerance)	(1) Red meat diet: diet high in lean red meat (≥200 g red meat/d for 6 d/week) with minimal dairy (<1 serving) (2) Dairy diet: a diet high in primarily low-fat dairy (4–6 servings/d, from milk, yogurt, or custard) with no red meat (3) Control diet: contained neither red meat nor dairy (≥200 g fish or chicken/d with <1 serving dairy/d)	3 × 4 weeks’ intervention; 2 weeks’ washout	HOMA-IR, Matsuda index, C-peptide, fasting glucose and insulin by oral-glucose-tolerance tests	Fasting insulin: higher after the dairy diet than after the red meat diet (*p* < 0.01), with no change in fasting glucose. Insulin resistance (HOMA-IR) increased (insulin sensitivity decreased) after the high-dairy diet (*p* < 0.05). Women: HOMA-IR was significantly higher and Matsuda insulin sensitivity was 14.7% lower after the dairy diet compared with the red meat diet (*p* < 0.01). No significant diet differences were observed in men. C-peptide did not differ between diets.
Milk—Fat Content and Comparisons with Other Beverages					
McDonald et al., 2019 [121]	n = 22 Prediabetes	3 intervention arms: (1) GLU group: 75 g glucose dissolved in 473 mL water (2) NFM group: 75 g glucose dissolved in 473 mL of non-fat dairy milk (3) FFM group: 75 g glucose dissolved in 473 mL full-fat dairy milk (3.25%)	180 min	Postprandial plasma glucose, insulin, iAUC glucose postprandial, iAUC0-180 min insulin, iAUC0-180 min lipids	Postprandial glycemia: NFM or FFM similarly attenuated postprandial glucose excursions compared to GLU alone (glucose iAUC0-180 min *p* < 0.05), with no difference between the two milk fat types; Insulin & Lipids: no significant differences between groups.
Li et al., 2017 [118]	n = 46 (n = 29 healthy and n = 17 Prediabetes)	Standard breakfast sandwich (422 kcal, 48 g carbs, 2 g fiber) with either 240 mL of one of six iso-volumetric breakfast beverages on separate mornings: (1) control: water (0 kcal, 0 g carbs) (2) Sugar-Sweetened Coffee (SSC): 50 kcal, 12 g carbs from sucrose (3) Reduced-Energy Orange Juice (Trop50®): 50 kcal, 12 g carbs from fructose, sucrose, glucose (4) Low-Fat Milk (LFM, 1% fat): 101 kcal, 12 g carbs from lactose, 8 g milk protein (5) Fat-Free Milk (FFM): 82 kcal, 12 g carbs from lactose, 8 g milk protein (6) Whole Milk (WM): 159 kcal, 12 g carbs from lactose, 9 g milk protein	240 min; ≥6 days’ washout	Postprandial plasma glucose and insulin concentrations, iAUC for 0–2 h, 2–4 h, and 0–4 h, and peak glucose and insulin concentrations; HOMA-IR, HOMA-beta, dietary intake, body composition	Beverage Comparison: iAUC0–4 h glucose was higher for SSC compared to water, orange juice, and LFM (*p* < 0.05); peak glucose higher for coffee compared to all other beverages (*p* < 0.05); iAUC0–4h insulin: higher for coffee and LFM vs. orange juice and water (*p* < 0.05). LFM showed late-phase insulinotrophic effect, yielding the highest insulin iAUC2–4 h (*p* < 0.05) Milk Fat Comparison: iAUC0–4 h glucose: did not differ between the water control and any of the milk types (FFM, LFM, WM).
Yogurt—Full-Fat Versus Non-Fat					
Taormina et al., 2025 [119]	n = 13 Prediabetes	Three daily servings of (1) FFY: full-fat yoghurt (3.25%, 170 g per 2000 kcal/d); macronutrient split: 15% protein, 38% fat, 47% carbohydrate or (2) NFY: non-fat yogurt; macronutrient split: 15% protein, 30% fat, 55% carbohydrate;	2 × 3 weeks; 2 weeks run-in	Primary outcome: glucose during 120 OGTT; secondary: fasting and postprandial glucose; fructosamine, insulin, C-peptide, HOMA-IR, QUICKI, Matsuda insulin sensitivity index; incretin hormones (GLP-1 and GIP)	No difference in glucose at 120 min OGTT (*p* = 0.9); FFY: lower fructosamine (211 ± 4 vs. 218 ± 4 µmol/L; *p* = 0.01); C-peptide (OGTT): 60 min value significantly lower in FFY (8.7 ± 0.6 vs. 9.4 ± 0.6 ng/mL; *p* = 0.01); greater fasting GLP-1 (*p* = 0.01) and post-prandial GIP (*p* = 0.01) in mixed meal tolerance test (MMTT)
Milk Formulations—Lactose Hydrolysis and Added Plant Extracts and Fibers					
Sun et al., 2025 [137]	n = 11 Prediabetes	4 test meals: Each beverage alongside 1 pack of whole-wheat bread (50 g carbs) (1) Control pure milk (PM): 220 mL (2) Lactose-hydrolyzed milk (LHM): 220 mL (3) LHM + Mulberry leaf extract (MLE) + corn silk extract (CSE) + Resistant Dextrin (RD): 220 mL (4) Galactooligosaccharide-enriched milk (GOS) + MLE + CSE: 220 mL	120; 7 days washout	Postprandial glucose response (iAUC, 1h PPG and 2 h PPG glucose, max. PPG, max. 2 h PPG, other in vitro	No statistically significant differences in PPGR parameters (such as AUC, 1 h PPG and 2 h PPG) across the four milk-based interventions when analyzing the entire group. Subgroup analysis with BMI > 24 kg/m^2^: significant reduction in postprandial parameters (1 h PPG, max. PPG and glucose excursion) with GOS + MLE + CSE vs. PM.
Yogurt—Added Heat-Treated L. Plantarum OLL2712 Versus Conventional Yogurt					
Toshimitsu et al., 2024 [112]	n = 148 Prediabetes (n = 145 completed/analyzed)	1x daily: 112 g of (1) *L. plantarum* OLL2712 (OLL2712): probiotic yogurt containing heat-treated *L. plantarum* OLL2712 (2) Placebo: conventional yogurt	12 weeks ingestion period; 8 weeks post-observation period	Primary outcome: HbA1c at week 12 and week 16 Secondary outcomes: GA, FPG, fasting serum insulin, HOMA-IR, serum cytokines, lipids, BMI, body weight	HbA1c: decrease greater in the OLL2712 group vs. placebo at week 12 (5.71 vs. 5.78%, *p* = 0.014) and week 16 (5.76 vs. 5.83%, *p* = 0.006); at week 20 HbA1c returned to baseline with no inter-group differences; GA: decreased in both groups at week 12; at week 16 GA remained decreased only in the OLL2712 group and showed a strong trend toward lower levels compared to placebo (*p* = 0.053) FPG: No changes in FPG in overall cohort; subgroup with elevated baseline FPG: reduction in FPG at week 8 vs. week 0 only in the OLL2712 group (*p* = 0.006); Subgroup with persistent elevated HbA1c: greater reduction in HbA1c in the OLL2712 group compared to placebo at week 12 (*p* = 0.006) and week 16 (*p* = 0.005) vs. placebo.
Toshimitsu et al., 2020 [113]	n = 130 Prediabetes (n = 126 completed/analyzed)	Daily consumption of (1) OLL2712: 112 g/d probiotic yogurt containing > 5 × 10^9^ heat-treated *L. plantarum* OLL2712 cells vs. (2) Placebo: 112 g/d conventional yogurt (fermented with standard cultures *L. bulgaricus* and *S. thermophilus*, but without OLL2712; nutrients: 56 kcal, 4.0 g protein, 1.6 g fat, 10.9 g carbohydrate)	4 weeks’ screening; 12 weeks’ intervention	Primary: HbA1c, FPG changes secondary: GA, fasting insulin, HOMA-IR, markers of inflammation, other	HbA1c reduction greater in OLL2712 group than placebo (−0.12 vs. −0.07%, *p* = 0.047). FBG did not change overall. Insulin: increased in the placebo group at week 12 compared to baseline (insulin change: +1.68 ± 4.03µU/mL, *p* < 0.01; HOMA-IR change: +0.42 ± 1.12, *p* < 0.05); GA: decreased similarly within both groups (Placebo: −0.2 ± 0.5% vs. OLL2712: −0.3 ± 0.5%, *p* < 0.01); Subgroup with elevated baseline FBG: FBG decreased significantly compared to baseline at week 4 (*p* < 0.05) and week 12 (*p* < 0.05) only in the OLL2712 group (FBG change: −2.8 ± 8.5 mg/dL).

AD, Adequate Dairy; AUC Area Under the Curve; BMI, Body Mass Index; cMax, Maximum Concentration; CSE, Corn Silk Extract; FFD; Full-Fat Dairy; FFM, Full-Fat Dairy Milk/Fat-Free Milk; FFY, Full-Fat Yoghurt; FPG, Fasting Plasma Glucose; FBG, Fasting Blood Glucose; GA, Gylcoalbumin; GIP, Glucose-dependent Insulinotropic Polypeptide; GLP-1, Glucagon-like Peptide 1; GOS, Galactooligosaccharide-Enriched Milk; HbA1c, Hemoglobin A1c; HD, High-Dose/High Dairy; HOMA-IR, Homeostasis Model Assessment of Insulin Resistance; iAUC, Incremental Area Under the Curve; ISI, Insulin Sensitivity Index; kcal, kilocalorie; LD, Low dose; LFM, Low-Fat Milk; LHM, Lactose-Hydrolyzed Milk; LMTT, Liquid Meal Tolerance Test; OGTT, Oral Glucose Tolerance Test; OLL2712, Lactiplantibacillus Plantarum OLL2712; MD19, Maltodextrin 19; MLE, Mulberry Leaf Extract; CSE, Corn Silk Extract; max, maximal; MMTT, Mixed Meal Tolerance Test; miRNA, MicroRNA; n, number; NFD, Non-Fat Dairy; NFM, Non-Fat Dairy Milk; NFY, Non-Fat Yoghurt; PM, Pure Milk; PPG, Postprandial Glucose; PPGR, Postprandial Glycemic Response; QUICKI, Quantitative Insulin Sensitivity Check Index; RD, Resistant Dextrin; SC, Sodium Caseinate; SSC, Sugar-Sweetened Coffee; SSP, Sugar-Sweetened Products; wk, week; WM, Whole Milk; WP, Whey Protein; WPI; Whey Protein Isolate.

## References

[1] Sanjulián L., Fernández-Rico S., González-Rodríguez N., Cepeda A., Miranda J.M., Fente C., Lamas A., Regal P. (2025). The Role of Dairy in Human Nutrition: Myths and Realities. Nutrients.

[2] Shkembi B., Huppertz T. (2023). Glycemic Responses of Milk and Plant-Based Drinks: Food Matrix Effects. Foods.

[3] Wang M., Wang Q., Chen B., Liu J. (2026). A narrative review of nutritional components, health effects, and disease prevention mechanisms of dairy products. Front. Public Health.

[4] Bjørnshave A., Hermansen K. (2014). Effects of Dairy Protein and Fat on the Metabolic Syndrome and Type 2 Diabetes. Rev. Diabet. Stud..

[5] Hidayat K., Du X., Shi B.M. (2019). Milk in the prevention and management of type 2 diabetes: The potential role of milk proteins. Diabetes/Metabolism Res. Rev..

[6] Sakata Y., Yago T., Mori S., Seto N., Matsunaga Y., Nakamura H., Tominaga T., Miyaji K., Takeda Y. (2022). Time Courses of Gastric Volume and Content after Different Types of Casein Ingestion in Healthy Men: A Randomized Crossover Study. J. Nutr..

[7] Asbaghi O., Khosroshahi M.Z., Kashkooli S., Abbasnezhad A. (2019). Effect of Calcium-Vitamin D Co-Supplementation on Insulin, Insulin Sensitivity, and Glycemia: A Systematic Review and Meta-Analysis of Randomized Clinical Trials. Horm. Metab. Res..

[8] Pittas A.G., Lau J., Hu F.B., Dawson-Hughes B. (2007). The Role of Vitamin D and Calcium in Type 2 Diabetes. A Systematic Review and Meta-Analysis. J. Clin. Endocrinol. Metab..

[9] Amrein K., Kim S.H., Brath H., Fasching P., Pittas A.G. (2026). Mini-review. Vitamin D for the prevention of type 2 diabetes: Evidence and implications. Metabolism.

[10] U.S. Department of Health and Human Services, U.S. Department of Agriculture (2026). Dietary Guidelines for Americans, 2025–2030.

[11] U.S. Department of Health and Human Services, U.S. Department of Agriculture (2026). The Scientific Foundation for the Dietary Guidelines for Americans, 2025–2030.

[12] American Diabetes Association Professional Practice Committee for Diabetes (2025). 2. Diagnosis and Classification of Diabetes: Standards of Care in Diabetes—2026. Diabetes Care.

[13] Samson S.L., Vellanki P., Blonde L., Hirsch I.B., Hoang T.D., Isaacs S.D., Izuora K.E., Wang C.C.L., Ooi C.P., Padilla B.I. (2026). American Association of Clinical Endocrinology Consensus Statement: Algorithm for Management of Adults with Type 2 Diabetes—2026 Update. Endocr. Pract..

[14] James D.E., Stöckli J., Birnbaum M.J. (2021). The aetiology and molecular landscape of insulin resistance. Nat. Rev. Mol. Cell Biol..

[15] Petersen M.C., Shulman G.I. (2018). Mechanisms of Insulin Action and Insulin Resistance. Physiol. Rev..

[16] Sperling L.S., Mechanick J.I., Neeland I.J., Herrick C.J., Després J.P., Ndumele C.E., Vijayaraghavan K., Handelsman Y., Puckrein G.A., Araneta M.R.G. (2015). The CardioMetabolic Health Alliance: Working Toward a New Care Model for the Metabolic Syndrome. J. Am. Coll. Cardiol..

[17] American Diabetes Association Professional Practice Committee for Diabetes (2025). 3. Prevention or Delay of Diabetes and Associated Comorbidities: Standards of Care in Diabetes—2026. Diabetes Care.

[18] Willett W.C., Ludwig D.S. (2020). Milk and Health. N. Engl. J. Med..

[19] Nijman R.M., Liu Y., Bunyatratchata A., Smilowitz J.T., Stahl B., Barile D. (2018). Characterization and Quantification of Oligosaccharides in Human Milk and Infant Formula. J. Agric. Food Chem..

[20] Isernhagen L., Galuska C.E., Vernunft A., Galuska S.P. (2024). Structural Characterization and Abundance of Sialylated Milk Oligosaccharides in Holstein Cows during Early Lactation. Foods.

[21] Robinson R.C. (2019). Structures and Metabolic Properties of Bovine Milk Oligosaccharides and Their Potential in the Development of Novel Therapeutics. Front. Nutr..

[22] German J.B., Dillard C.J. (2006). Composition, Structure and Absorption of Milk Lipids: A Source of Energy, Fat-Soluble Nutrients and Bioactive Molecules. Crit. Rev. Food Sci. Nutr..

[23] Yao D., Ranadheera C.S., Shen C., Wei W., Cheong L.Z. (2024). Milk fat globule membrane: Composition, production and its potential as encapsulant for bioactives and probiotics. Crit. Rev. Food Sci. Nutr..

[24] Unger A.L., Bourne D.E., Walsh H., Kraft J. (2020). Fatty Acid Content of Retail Cow’s Milk in the Northeastern United States—What’s in It for the Consumer?. J. Agric. Food Chem..

[25] Frizzarin M., O’Callaghan T.F., Berry D.P. (2025). Factors associated with the concentration of fatty acids in the milk from grazing dairy cows. J. Dairy Sci..

[26] Auestad N., Layman D.K. (2021). Dairy bioactive proteins and peptides: A narrative review. Nutr. Rev..

[27] Toffolon A., de Rocco-Ponce M., Vettore M., Iori E., Lante A., Tessari P. (2021). Effect of Reversal of Whey-Protein to Casein Ratio of Cow Milk, on Insulin, Incretin, and Amino Acid Responses in Humans. Mol. Nutr. Food Res..

[28] Gaucheron F. (2011). Milk and Dairy Products: A Unique Micronutrient Combination. J. Am. Coll. Nutr..

[29] Magan J.B., O Callaghan T.F., Kelly A.L., McCarthy N.A. (2021). Compositional and functional properties of milk and dairy products derived from cows fed pasture or concentrate-based diets. Compr. Rev. Food Sci. Food Saf..

[30] Valentino V., Magliulo R., Farsi D., Cotter P.D., O’Sullivan O., Ercolini D., De Filippis F. (2024). Fermented foods, their microbiome and its potential in boosting human health. Microb. Biotechnol..

[31] Alm L. (1982). Effect of fermentation on lactose, glucose, and galactose content in milk and suitability of fermented milk products for lactose intolerant individuals. J. Dairy Sci..

[32] Harper A.R., Dobson R.C.J., Morris V.K., Moggré G.J. (2022). Fermentation of plant-based dairy alternatives by lactic acid bacteria. Microb. Biotechnol..

[33] Salari A., Ghodrat S., Gheflati A., Jarahi L., Hashemi M., Afshari A. (2021). Effect of kefir beverage consumption on glycemic control: A systematic review and meta-analysis of randomized controlled clinical trials. Complement. Ther. Clin. Pract..

[34] Fernández M., Hudson J.A., Korpela R., de Los Reyes-Gavilán C.G. (2015). Impact on human health of microorganisms present in fermented dairy products: An overview. BioMed Res. Int..

[35] Marco M.L., Sanders M.E., Gänzle M., Arrieta M.C., Cotter P.D., De Vuyst L., Hill C., Holzapfel W., Lebeer S., Merenstein D. (2021). The International Scientific Association for Probiotics and Prebiotics (ISAPP) consensus statement on fermented foods. Nat. Rev. Gastroenterol. Hepatol..

[36] Kumar S., Rai D.C., Niranjan K., Bhat Z.F. (2014). Paneer—An Indian soft cheese variant: A review. J. Food Sci. Technol..

[37] Mangione G., Caccamo M., Natalello A., Licitra G. (2023). Graduate Student Literature Review: History, technologies of production, and characteristics of ricotta cheese. J. Dairy Sci..

[38] Sharma H., Ozogul F., Bartkiene E., Rocha J.M. (2023). Impact of lactic acid bacteria and their metabolites on the techno-functional properties and health benefits of fermented dairy products. Crit. Rev. Food Sci. Nutr..

[39] Gibson G.R., Hutkins R., Sanders M.E., Prescott S.L., Reimer R.A., Salminen S.J., Scott K., Stanton C., Swanson K.S., Cani P.D. (2017). Expert consensus document: The International Scientific Association for Probiotics and Prebiotics (ISAPP) consensus statement on the definition and scope of prebiotics. Nat. Rev. Gastroenterol. Hepatol..

[40] Cooper T.E., Khalid R., Chan S., Craig J.C., Hawley C.M., Howell M., Johnson D.W., Jaure A., Teixeira-Pinto A., Wong G. Synbiotics, Prebiotics and Probiotics for People with Chronic Kidney Disease-Cooper, TE-2023|Cochrane Library [Internet]. https://www.cochranelibrary.com/cdsr/doi/10.1002/14651858.CD013631.pub2/full.

[41] Zepeda-Hernández A., Garcia-Amezquita L.E., Requena T., García-Cayuela T. (2021). Probiotics, prebiotics, and synbiotics added to dairy products: Uses and applications to manage type 2 diabetes. Food Res. Int..

[42] Joint FAO/WHO Working Group (2002). Report on Drafting Guidelines for the Evaluation of Probiotics in Food. Guidelines for the Evaluation of Probiotics in Food. [Internet]. London, Ontario, Canada. https://isappscience.org/wp-content/uploads/2019/04/probiotic_guidelines.pdf.

[43] Cremon C., Barbaro M.R., Ventura M., Barbara G. (2018). Pre- and probiotic overview. Curr. Opin. Pharmacol..

[44] McCarthy K.S., Lopetcharat K., Drake M.A. (2017). Milk fat threshold determination and the effect of milk fat content on consumer preference for fluid milk. J. Dairy Sci..

[45] Jensen R.G., Ferris A.M., Lammi-Keefe C.J. (1991). The composition of milk fat. J. Dairy Sci..

[46] Hirahatake K.M., Bruno R.S., Bolling B.W., Blesso C., Alexander L.M., Adams S.H. (2020). Dairy Foods and Dairy Fats: New Perspectives on Pathways Implicated in Cardiometabolic Health. Adv. Nutr..

[47] Hirahatake K.M., Astrup A., Hill J.O., Slavin J.L., Allison D.B., Maki K.C. (2020). Potential Cardiometabolic Health Benefits of Full-Fat Dairy: The Evidence Base. Adv. Nutr..

[48] Lichtenstein A.H., Khera A., Anderson C.A.M., Appel L.J., DeSilva D.M., Gardner C., Hu F.B., Jones D.W., Petersen K.S. (2026). 2026 Dietary Guidance to Improve Cardiovascular Health: A Scientific Statement From the American Heart Association. Circulation.

[49] Lamarche B., Astrup A., Eckel R.H., Feeney E., Givens I., Krauss R.M., Legrand P., Micha R., Michalski M.-C., Soedamah-Muthu S. (2025). Regular-fat and low-fat dairy foods and cardiovascular diseases: Perspectives for future dietary recommendations. Am. J. Clin. Nutr..

[50] Arnesen E.K., Christensen J.J., Laake I., Carlsen M.H., Veierød M.B., Retterstøl K. (2025). Low-fat and whole milk consumption in relation to cardiovascular disease–related and all-cause mortality: A prospective cohort study in 3 Norwegian counties. Am. J. Clin. Nutr..

[51] Oberoi A., Giezenaar C., Rigda R.S., Horowitz M., Jones K.L., Chapman I., Soenen S. (2023). Effects of co-ingesting glucose and whey protein on blood glucose, plasma insulin and glucagon concentrations, and gastric emptying, in older men with and without type 2 diabetes. Diabetes Obes. Metab..

[52] Dao G.M., Shaw C.S., Betik A.C., Kuriel V., Bruce C.R., Kowalski G.M. (2025). Mechanistic Insights Into Postprandial Insulin-Glucagon Interactions and Their Impact on Glucose Flux After Protein-Glucose Coingestion in Humans. Diabetes.

[53] Vimaleswaran K.S., Mohan V. (2026). Dairy intake and risk of type 2 diabetes and metabolic syndrome: A narrative review. Nutrition.

[54] Gijsbers L., Ding E.L., Malik V.S., de Goede J., Geleijnse J.M., Soedamah-Muthu S.S. (2016). Consumption of dairy foods and diabetes incidence: A dose-response meta-analysis of observational studies. Am. J. Clin. Nutr..

[55] Alvarez-Bueno C., Cavero-Redondo I., Martinez-Vizcaino V., Sotos-Prieto M., Ruiz J.R., Gil A. (2019). Effects of Milk and Dairy Product Consumption on Type 2 Diabetes: Overview of Systematic Reviews and Meta-Analyses. Adv. Nutr..

[56] Lordan R. (2024). A new era for food in health? The FDA announces a qualified health claim for yogurt intake and type 2 diabetes mellitus risk reduction. Diabetes Metab. Syndr..

[57] Salas-Salvadó J., Guasch-Ferré M., Díaz-López A., Babio N. (2017). Yogurt and Diabetes: Overview of Recent Observational Studies. J. Nutr..

[58] Aune D., Norat T., Romundstad P., Vatten L.J. (2013). Dairy products and the risk of type 2 diabetes: A systematic review and dose-response meta-analysis of cohort studies123. Am. J. Clin. Nutr..

[59] Zhang S., Janzi S., Du Y., Smith J.G., Qi L., Borné Y., Sonestedt E. (2025). Dairy intake, plasma metabolome, and risk of type 2 diabetes in a population-based cohort. Am. J. Clin. Nutr..

[60] Drouin-Chartier J.P., Li Y., Ardisson Korat A.V., Ding M., Lamarche B., Manson J.E., Rimm E.B., Willett W.C., Hu F.B. (2019). Changes in dairy product consumption and risk of type 2 diabetes: Results from 3 large prospective cohorts of US men and women. Am. J. Clin. Nutr..

[61] Hruby A., Ma J., Rogers G., Meigs J.B., Jacques P.F. (2017). Associations of Dairy Intake with Incident Prediabetes or Diabetes in Middle-Aged Adults Vary by Both Dairy Type and Glycemic Status. J. Nutr..

[62] Zhang M., Dong X., Huang Z., Li X., Zhao Y., Wang Y., Zhu H., Fang A., Giovannucci E.L. (2023). Cheese consumption and multiple health outcomes: An umbrella review and updated meta-analysis of prospective studies. Adv. Nutr..

[63] Yang Y., Wang X., Yang W. (2026). Examining the causal link between cheese intake and susceptibility to type 2 diabetes through metabolic traits: Insights from Mendelian randomization. J. Dairy Sci..

[64] Brouwer-Brolsma E.M., Sluik D., Singh-Povel C.M., Feskens E.J.M. (2018). Dairy product consumption is associated with pre-diabetes and newly diagnosed type 2 diabetes in the Lifelines Cohort Study. Br. J. Nutr..

[65] Slurink I.A., Corpeleijn E., Bakker S.J., Jongerling J., Kupper N., Smeets T., Soedamah-Muthu S.S. (2023). Dairy consumption and incident prediabetes: Prospective associations and network models in the large population-based Lifelines Study. Am. J. Clin. Nutr..

[66] Slurink I.A., Chen L., Magliano D.J., Kupper N., Smeets T., Soedamah-Muthu S.S. (2023). Dairy Product Consumption and Incident Prediabetes in the Australian Diabetes, Obesity, and Lifestyle Study with 12 Years of Follow-Up. J. Nutr..

[67] Slurink I.A.L., Kupper N., Smeets T., Soedamah-Muthu S.S. (2024). Dairy consumption and risk of prediabetes and type 2 diabetes in the Fenland study. Clin. Nutr..

[68] Díaz-López A., Bulló M., Martínez-González M.A., Corella D., Estruch R., Fitó M., Gómez-Gracia E., Fiol M., Garcia De La Corte F.J., Ros E. (2016). Dairy product consumption and risk of type 2 diabetes in an elderly Spanish Mediterranean population at high cardiovascular risk. Eur. J. Nutr..

[69] Bhavadharini B., Dehghan M., Mente A., Rangarajan S., Sheridan P., Mohan V., Iqbal R., Gupta R., Lear S., Wentzel-Viljoen E. (2020). Association of dairy consumption with metabolic syndrome, hypertension and diabetes in 147 812 individuals from 21 countries. BMJ Open Diabetes Res. Care.

[70] Tsameret S., Froy O., Matz Y., Landau Z., Twito O., Wainstein J., Avital-Cohen N., Chapnik N., Jakubowicz D. (2026). Glycaemic, appetite and circadian benefits of a dairy-enriched diet with high-protein breakfast and early daytime-restricted carbohydrate intake in type 2 diabetes: A randomised crossover trial. Diabetologia.

[71] Costa J.d.A., Gomes J.M.G., Ribeiro P.V.d.M., Alfenas R.d.C.G. (2020). Increased consumption of calcium from fat-free milk, energy-restricted diet and educational activities improves metabolic control in overweight type 2 diabetic patients. Br. J. Nutr..

[72] Cai X., Yu H., Liu L., Lu T., Li J., Ji Y., Le Z., Bao L., Ma W., Xiao R. (2018). Milk Powder Co-Supplemented with Inulin and Resistant Dextrin Improves Glycemic Control and Insulin Resistance in Elderly Type 2 Diabetes Mellitus: A 12-Week Randomized, Double-Blind, Placebo-Controlled Trial. Mol. Nutr. Food Res..

[73] Jakubowicz D., Wainstein J., Landau Z., Ahren B., Barnea M., Bar-Dayan Y., Froy O. (2017). High-energy breakfast based on whey protein reduces body weight, postprandial glycemia and HbA1C in Type 2 diabetes. J. Nutr. Biochem..

[74] Smith K., Taylor G.S., Walker M., Brunsgaard L.H., Bowden Davies K.A., Stevenson E.J., West D.J. (2023). Pre-Meal Whey Protein Alters Postprandial Insulinemia by Enhancing β-Cell Function and Reducing Insulin Clearance in T2D. J. Clin. Endocrinol. Metab..

[75] Wu T., Little T.J., Bound M.J., Borg M., Zhang X., Deacon C.F., Horowitz M., Jones K.L., Rayner C.K. (2016). A Protein Preload Enhances the Glucose-Lowering Efficacy of Vildagliptin in Type 2 Diabetes. Diabetes Care.

[76] King D.G., Walker M., Campbell M.D., Breen L., Stevenson E.J., West D.J. (2018). A small dose of whey protein co-ingested with mixed-macronutrient breakfast and lunch meals improves postprandial glycemia and suppresses appetite in men with type 2 diabetes: A randomized controlled trial. Am. J. Clin. Nutr..

[77] Jakubowicz D., Froy O., Ahrén B., Boaz M., Landau Z., Bar-Dayan Y., Ganz T., Barnea M., Wainstein J. (2014). Incretin, insulinotropic and glucose-lowering effects of whey protein pre-load in type 2 diabetes: A randomised clinical trial. Diabetologia.

[78] Tessari P., Kiwanuka E., Cristini M., Zaramella M., Enslen M., Zurlo C., Garcia-Rodenas C. (2007). Slow versus fast proteins in the stimulation of beta-cell response and the activation of the entero-insular axis in type 2 diabetes. Diabetes/Metab. Res. Rev..

[79] Mitri J., Tomah S., Mottalib A., Salsberg V., Ashrafzadeh S., Pober D.M., Eldib A.H., Tasabehji M.W., Hamdy O. (2020). Effect of dairy consumption and its fat content on glycemic control and cardiovascular disease risk factors in patients with type 2 diabetes: A randomized controlled study. Am. J. Clin. Nutr..

[80] Miraghajani M.S., Najafabadi M.M., Surkan P.J., Esmaillzadeh A., Mirlohi M., Azadbakht L. (2013). Soy Milk Consumption and Blood Pressure Among Type 2 Diabetic Patients with Nephropathy. J. Ren. Nutr..

[81] Gobert C.P., Pipe E.A., Capes S.E., Darlington G.A., Lampe J.W., Duncan A.M. (2010). Soya protein does not affect glycaemic control in adults with type 2 diabetes. Br. J. Nutr..

[82] Miller E.G., Nowson C.A., Dunstan D.W., Kerr D.A., Menzies D., Daly R.M. (2021). Effects of whey protein plus vitamin D supplementation combined with progressive resistance training on glycaemic control, body composition, muscle function and cardiometabolic risk factors in middle-aged and older overweight/obese adults with type 2 diabetes: A 24-week randomized controlled trial. Diabetes Obes. Metab..

[83] Soares A.d.S., Machado-Lima A., Brech G.C., Greve J.M.D., dos Santos J.R., Inojossa T.R., Rogero M.M., Salles J.E.N., Santarem-Sobrinho J.M., Davis C.L. (2023). The Influence of Whey Protein on Muscle Strength, Glycemic Control and Functional Tasks in Older Adults with Type 2 Diabetes Mellitus in a Resistance Exercise Program: Randomized and Triple Blind Clinical Trial. Int. J. Environ. Res. Public Health.

[84] Gaffney K.A., Lucero A., Stoner L., Faulkner J., Whitfield P., Krebs J., Rowlands D.S. (2018). Nil Whey Protein Effect on Glycemic Control after Intense Mixed-Mode Training in Type 2 Diabetes. Med. Sci. Sports Exerc..

[85] Gomes J.M.G., Costa J.d.A., Ribeiro P.d.M., Alfenas R., de C.G. (2019). High calcium intake from fat-free milk, body composition and glycaemic control in adults with type 2 diabetes: A randomised crossover clinical trial. Br. J. Nutr..

[86] Nikooyeh B., Neyestani T.R., Farvid M., Alavi-Majd H., Houshiarrad A., Kalayi A., Shariatzadeh N., Gharavi A., Heravifard S., Tayebinejad N. (2011). Daily consumption of vitamin D– or vitamin D + calcium–fortified yogurt drink improved glycemic control in patients with type 2 diabetes: A randomized clinical trial. Am. J. Clin. Nutr..

[87] Nikooyeh B., Hollis B.W., Neyestani T.R. (2021). The effect of daily intake of vitamin D-fortified yogurt drink, with and without added calcium, on serum adiponectin and sirtuins 1 and 6 in adult subjects with type 2 diabetes. Nutr. Diabetes.

[88] Jafari T., Faghihimani E., Feizi A., Iraj B., Javanmard S.H., Esmaillzadeh A., Fallah A.A., Askari G. (2016). Effects of vitamin D-fortified low fat yogurt on glycemic status, anthropometric indexes, inflammation, and bone turnover in diabetic postmenopausal women: A randomised controlled clinical trial. Clin. Nutr..

[89] Shab-Bidar S., Neyestani T.R., Djazayery A., Eshraghian M.R., Houshiarrad A., Gharavi A., Kalayi A., Shariatzadeh N., Zahedirad M., Khalaji N. (2011). Regular consumption of vitamin D-fortified yogurt drink (Doogh) improved endothelial biomarkers in subjects with type 2 diabetes: A randomized double-blind clinical trial. BMC Med..

[90] Hajimohammadi M., Shab-Bidar S., Neyestani T.R. (2017). Consumption of vitamin D-fortified yogurt drink increased leptin and ghrelin levels but reduced leptin to ghrelin ratio in type 2 diabetes patients: A single blind randomized controlled trial. Eur. J. Nutr..

[91] Salehi S., Sadeghi F., Akhlaghi M., Hanifpour M.A., Roshanzamir M. (2018). Vitamin D3-fortified milk did not affect glycemic control, lipid profile, and anthropometric measures in patients with type 2 diabetes, a triple-blind randomized clinical trial. Eur. J. Clin. Nutr..

[92] Yanni A.E., Kokkinos A., Psychogiou G., Binou P., Kartsioti K., Chatzigeorgiou A., Konstantopoulos P., Perrea D., Tentolouris N., Karathanos V.T. (2019). Daily consumption of fruit-flavored yoghurt enriched with vitamins B contributes to lower energy intake and body weight reduction, in type 2 diabetic patients: A randomized clinical trial. Food Funct..

[93] Mirjalili M., Salari Sharif A., Sangouni A.A., Emtiazi H., Mozaffari-Khosravi H. (2023). Effect of probiotic yogurt consumption on glycemic control and lipid profile in patients with type 2 diabetes mellitus: A randomized controlled trial. Clin. Nutr. ESPEN.

[94] Tonucci L.B., Olbrich Dos Santos K.M., Licursi De Oliveira L., Rocha Ribeiro S.M., Duarte Martino H.S. (2017). Clinical application of probiotics in type 2 diabetes mellitus: A randomized, double-blind, placebo-controlled study. Clin. Nutr..

[95] Ejtahed H.S., Mohtadi-Nia J., Homayouni-Rad A., Niafar M., Asghari-Jafarabadi M., Mofid V. (2012). Probiotic yogurt improves antioxidant status in type 2 diabetic patients. Nutrition.

[96] Sato J., Kanazawa A., Azuma K., Ikeda F., Goto H., Komiya K., Kanno R., Tamura Y., Asahara T., Takahashi T. (2017). Probiotic reduces bacterial translocation in type 2 diabetes mellitus: A randomised controlled study. Sci. Rep..

[97] Hove K.D., Brøns C., Færch K., Lund S.S., Rossing P., Vaag A. (2015). Effects of 12 weeks of treatment with fermented milk on blood pressure, glucose metabolism and markers of cardiovascular risk in patients with type 2 diabetes: A randomised double-blind placebo-controlled study. Eur. J. Endocrinol..

[98] Zhang C., Zhang Q., Zhang X., Du S., Zhang Y., Wang X., Liu Y., Fang B., Chen J., Liu R. (2025). Effects of synbiotics surpass probiotics alone in improving type 2 diabetes mellitus: A randomized, double-blind, placebo-controlled trial. Clin. Nutr..

[99] Ma J., Jesudason D.R., Stevens J.E., Keogh J.B., Jones K.L., Clifton P.M., Horowitz M., Rayner C.K. (2015). Sustained effects of a protein ‘preload’ on glycaemia and gastric emptying over 4 weeks in patients with type 2 diabetes: A randomized clinical trial. Diabetes Res. Clin. Pract..

[100] Derosa G., D’Angelo A., Maffioli P. (2020). Change of some oxidative stress parameters after supplementation with whey protein isolate in patients with type 2 diabetes. Nutrition.

[101] Smith K., Taylor G.S., Brunsgaard L.H., Walker M., Bowden Davies K.A., Stevenson E.J., West D.J. (2022). Thrice daily consumption of a novel, premeal shot containing a low dose of whey protein increases time in euglycemia during 7 days of free-living in individuals with type 2 diabetes. BMJ Open Diabetes Res. Care.

[102] Smedegaard S.B., Mose M., Hulman A., Mikkelsen U.R., Møller N., Wegener G., Jessen N., Rittig N. (2021). Β-Lactoglobulin Elevates Insulin and Glucagon Concentrations Compared with Whey Protein—A Randomized Double-Blinded Crossover Trial in Patients with Type Two Diabetes Mellitus. Nutrients.

[103] Pabla P., Mallinson J., Nixon A., Keeton M., Cooper S., Marshall M., Jacques M., Brown S., Johansen O.E., Cuenoud B. (2025). Effect of medium-chain triglycerides and whey protein isolate preloads on glycaemia in type 2 diabetes: A randomized crossover study. Am. J. Clin. Nutr..

[104] Sridonpai P., Prachansuwan A., Praengam K., Tuntipopipat S., Kriengsinyos W. (2021). Postprandial effects of a whey protein-based multi-ingredient nutritional drink compared with a normal breakfast on glucose, insulin, and active GLP-1 response among type 2 diabetic subjects: A crossover randomised controlled trial. J. Nutr. Sci..

[105] Sun Y., Zhang Y., Niu X., Liu Y., Xu L., Wang Y., Zhang Q., He J., Liu Y., Zhang K. (2025). Postprandial glycemic effects of lactose-hydrolyzed milk supplemented with mulberry leaf and corn silk extracts in adults with type 2 diabetes: A randomized crossover trial. Clin. Nutr. ESPEN.

[106] Dhaver S., Al-Badri M., Mitri J., Barbar Askar A.A., Mottalib A., Hamdy O. (2025). Effect of Almond Milk Versus Cow Milk on Postprandial Glycemia, Lipidemia, and Gastrointestinal Hormones in Patients with Overweight or Obesity and Type 2 Diabetes: A Randomized Controlled Clinical Trial. Nutrients.

[107] Bukar J., Mezitis N.H.E., Saitas V., Pi-Sunyer F.X. (1990). Frozen Desserts and Glycemic Response in Well-Controlled NIDDM Patients. Diabetes Care.

[108] Mortensen L.S., Holmer-Jensen J., Hartvigsen M.L., Jensen V.K., Astrup A., de Vrese M., Holst J.J., Thomsen C., Hermansen K. (2012). Effects of different fractions of whey protein on postprandial lipid and hormone responses in type 2 diabetes. Eur. J. Clin. Nutr..

[109] Bjørnshave A., Hermansen K., Holst J.J. (2018). Pre-Meal Effect of Whey Proteins on Metabolic Parameters in Subjects with and without Type 2 Diabetes: A Randomized, Crossover Trial. Nutrients.

[110] Wildová E., Kraml P., Potočková J., Dlouhý P., Anděl M. (2017). The Assessment of the Serum C-Peptide and Plasma Glucose Levels by Orally Administered Whey Proteins in Type 2 Diabetes Mellitus. Physiol. Res..

[111] Ang M., Müller A.S., Wagenlehner F., Pilatz A., Linn T. (2012). Combining protein and carbohydrate increases postprandial insulin levels but does not improve glucose response in patients with type 2 diabetes. Metabolism.

[112] Toshimitsu T., Gotou A., Sashihara T., Hojo K., Hachimura S., Shioya N., Iwama Y., Irie J., Ichihara Y. (2024). Ingesting probiotic yogurt containing Lactiplantibacillus plantarum OLL2712 improves glycaemic control in adults with prediabetes in a randomized, double-blind, placebo-controlled trial. Diabetes Obes. Metab..

[113] Toshimitsu T., Gotou A., Sashihara T., Hachimura S., Shioya N., Suzuki S., Asami Y. (2020). Effects of 12-Week Ingestion of Yogurt Containing Lactobacillus plantarum OLL2712 on Glucose Metabolism and Chronic Inflammation in Prediabetic Adults: A Randomized Placebo-Controlled Trial. Nutrients.

[114] Sartorius T., Weidner A., Dharsono T., Boulier A., Wilhelm M., Schön C. (2019). Postprandial Effects of a Proprietary Milk Protein Hydrolysate Containing Bioactive Peptides in Prediabetic Subjects. Nutrients.

[115] Hoefle A.S., Bangert A.M., Stamfort A., Gedrich K., Rist M.J., Lee Y.M., Skurk T., Daniel H. (2015). Metabolic Responses of Healthy or Prediabetic Adults to Bovine Whey Protein and Sodium Caseinate Do Not Differ. J. Nutr..

[116] Bjørnshave A., Holst J.J., Hermansen K. (2019). A pre-meal of whey proteins induces differential effects on glucose and lipid metabolism in subjects with the metabolic syndrome: A randomised cross-over trial. Eur. J. Nutr..

[117] Turner K.M., Keogh J.B., Clifton P.M. (2015). Red meat, dairy, and insulin sensitivity: A randomized crossover intervention study. Am. J. Clin. Nutr..

[118] Li J., Janle E., Campbell W.W. (2017). Postprandial Glycemic and Insulinemic Responses to Common Breakfast Beverages Consumed with a Standard Meal in Adults Who Are Overweight and Obese. Nutrients.

[119] Taormina V.M., Eisenhardt S., Gilbert M.P., Poynter M.E., Kien C.L., Kraft J. (2025). Full-fat versus non-fat yogurt consumption improves glucose homeostasis and metabolic hormone regulation in individuals with prediabetes: A randomized-controlled trial. Nutr. Res..

[120] McDonald J.D., Mah E., Chitchumroonchokchai C., Dey P., Labyk A.N., Villamena F.A., Volek J.S., Bruno R.S. (2019). Dairy milk proteins attenuate hyperglycemia-induced impairments in vascular endothelial function in adults with prediabetes by limiting increases in glycemia and oxidative stress that reduce nitric oxide bioavailability. J. Nutr. Biochem..

[121] McDonald J.D., Mah E., Dey P., Olmstead B.D., Sasaki G.Y., Villamena F.A., Bruno R.S. (2019). Dairy milk, regardless of fat content, protects against postprandial hyperglycemia-mediated impairments in vascular endothelial function in adults with prediabetes by limiting oxidative stress responses that reduce nitric oxide bioavailability. J. Nutr. Biochem..

[122] American Diabetes Association Professional Practice Committee for Diabetes (2025). 9. Pharmacologic Approaches to Glycemic Treatment: Standards of Care in Diabetes—2026. Diabetes Care.

[123] Taormina V.M., Eisenhardt S., Gilbert M.P., Poynter M.E., Kien C.L., Kraft J. (2025). Full-fat yogurt compared with non-fat yogurt reduces blood triacylglycerol concentrations and lowers the triacylglycerol content in specific lipoprotein subclasses in adults with prediabetes: An exploratory analysis of a randomized-controlled trial. Lipids Health Dis..

[124] O’Connor S., Turcotte A.F., Gagnon C., Rudkowska I. (2019). Increased Dairy Product Intake Modifies Plasma Glucose Concentrations and Glycated Hemoglobin: A Systematic Review and Meta-Analysis of Randomized Controlled Trials. Adv. Nutr..

[125] Sochol K.M., Johns T.S., Buttar R.S., Randhawa L., Sanchez E., Gal M., Lestrade K., Merzkani M., Abramowitz M.K., Mossavar-Rahmani Y. (2019). The Effects of Dairy Intake on Insulin Resistance: A Systematic Review and Meta-Analysis of Randomized Clinical Trials. Nutrients.

[126] Companys J., Pla-Pagà L., Calderón-Pérez L., Llauradó E., Solà R., Pedret A., Valls R.M. (2020). Fermented Dairy Products, Probiotic Supplementation, and Cardiometabolic Diseases: A Systematic Review and Meta-analysis. Adv. Nutr..

[127] Feng Y., Zhao Y., Liu J., Huang Z., Yang X., Qin P., Chen C., Luo X., Li Y., Wu Y. (2022). Consumption of Dairy Products and the Risk of Overweight or Obesity, Hypertension, and Type 2 Diabetes Mellitus: A Dose–Response Meta-Analysis and Systematic Review of Cohort Studies. Adv. Nutr..

[128] Smedegaard S., Kampmann U., Ovesen P.G., Støvring H., Rittig N. (2023). Whey Protein Premeal Lowers Postprandial Glucose Concentrations in Adults Compared with Water-The Effect of Timing, Dose, and Metabolic Status: A Systematic Review and Meta-analysis. Am. J. Clin. Nutr..

[129] Nilsson M., Holst J.J., Björck I.M. (2007). Metabolic effects of amino acid mixtures and whey protein in healthy subjects: Studies using glucose-equivalent drinks2. Am. J. Clin. Nutr..

[130] Ma J., Stevens J.E., Cukier K., Maddox A.F., Wishart J.M., Jones K.L., Clifton P.M., Horowitz M., Rayner C.K. (2009). Effects of a Protein Preload on Gastric Emptying, Glycemia, and Gut Hormones After a Carbohydrate Meal in Diet-Controlled Type 2 Diabetes. Diabetes Care.

[131] Watson L.E., Phillips L.K., Wu T., Bound M.J., Checklin H.L., Grivell J., Jones K.L., Clifton P.M., Horowitz M., Rayner C.K. (2019). A whey/guar “preload” improves postprandial glycaemia and glycated haemoglobin levels in type 2 diabetes: A 12-week, single-blind, randomized, placebo-controlled trial. Diabetes Obes. Metab..

[132] Genitsaridi I., Salpea P., Salim A., Sajjadi S.F., Tomic D., James S., Thirunavukkarasu S., Issaka A., Chen L., Basit A. (2026). 11th edition of the IDF Diabetes Atlas: Global, regional, and national diabetes prevalence estimates for 2024 and projections for 2050. Lancet Diabetes Endocrinol..

[133] Milton J.E., Sananthanan C.S., Patterson M., A Ghatei M., Bloom S.R., Frost G.S. (2007). Glucagon-like peptide-1 (7-36) amide response to low versus high glycaemic index preloads in overweight subjects with and without type II diabetes mellitus. Eur. J. Clin. Nutr..

[134] Gomes J.M.G., Costa J.D.A., Alfenas R.C.G. (2018). Effect of increased calcium consumption from fat-free milk in an energy-restricted diet on the metabolic syndrome and cardi-ometabolic outcomes in adults with type 2 diabetes mellitus: A randomised cross-over clinical trial. Br. J. Nutr..

[135] Nikooyeh B., Shariatzadeh N., Rismanchi M., Hollis B.W., Neyestani T.R. (2021). Daily intake of yogurt drink fortified either with vitamin D alone or in combination with added calcium causes a thyroid-independent increase of resting metabolic rate in adults with type 2 diabetes: A randomized, double-blind, clinical trial. Appl. Physiol. Nutr. Metab..

[136] Shab-Bidar S., Neyestani T.R., Djazayery A. (2015). Vitamin D receptor Cdx-2-dependent response of central obesity to vitamin D intake in the subjects with type 2 diabetes: A randomised clinical trial. Br. J. Nutr..

[137] Sun Y., Niu X., Wang Y., Zhang Q., Liu Y., He J., Xu L., Wang R., Guo J. (2025). Effects of Mulberry Leaf and Corn Silk Extracts Against α-Amylase and α-Glucosidase In Vitro and on Postprandial Glucose in Prediabetic Individuals: A Randomized Crossover Trial. Nutrients.

